# Recent Insights in Multi‐Target Drugs in Pharmacology and Medicinal Chemistry

**DOI:** 10.1002/cmdc.202500447

**Published:** 2025-09-01

**Authors:** Sadık Hüseyin Cemali, Samet Poyraz, Samet Belveren, Senanur Taş, Mehmet Ali Tamer, Naciye Yaktubay Döndaş, H. Ali Döndaş, Jose Miguel Sansano

**Affiliations:** ^1^ Faculty of Pharmacy Department of Basic Pharmaceutical Sciences Çukurova University Balcalı 01330 Adana Türkiye; ^2^ Faculty of Pharmacy Department of Analytical Chemistry Ağrı İbrahim Çeçen University 04100 Ağrı Türkiye; ^3^ Faculty of Pharmacy Department of Analytical Chemistry Mersin University 33169 Mersin Türkiye; ^4^ Department of Biotechnology Institute of Natural and Applied Sciences Çukurova University Balcalı 01330 Adana Türkiye; ^5^ Faculty of Medicine Department of Medical Pharmacology Cukurova University 01330 Adana Türkiye; ^6^ Department of Translational Medicine Institute of Health Sciences Çukurova University 01330 Adana Türkiye; ^7^ Department of Organic Chemistry Centro de Innovación en Química Avanzada (ORFEO‐CINQA) and Instituto de Síntesis Orgánica (ISO) University of Alicante 03690 Alicante Spain

**Keywords:** cancer, molecular docking, multitargeted drugs, neurodegenerative diseases, synthesis methods

## Abstract

Many of the drugs used in treatment today have been designed based on the “specificity paradigm”. Resistance has developed against drugs designed using this approach, leading to a decrease in their effectiveness. In addition, it is well‐documented in the literature that diseases with complex etiologies, such as Alzheimer's, Parkinson's, and cancer are influenced by multiple genetic and/or environmental factors. As a result, specificity paradigm is often insufficient for treating these diseases. Therefore, there is a need to develop drugs that interact with multiple targets simultaneously through different mechanisms. This review aims to provide an overview of the methods used in multitarget drug design, the reactions employed in the synthesis of these drugs, their applications, and recent research conducted in this field.

## Graphical abstract

This review highlights the rationale behind multitarget drug design as a promising approach to address diseases with complex etiologies. By combining pharmacophore features from different single‐target drugs, multitarget compounds can interact with multiple biological targets simultaneously. This strategy offers improved therapeutic outcomes, reduces the likelihood of resistance, and enhances synergistic or additive effects compared to traditional single‐target approaches.

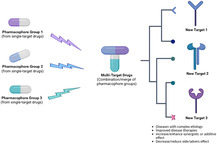

## 1 Introduction

Multitarget drugs are molecules that incorporate pharmacophore groups for two or more biological targets, which may share similar or distinct mechanisms of action at different molecular binding sites, within a single structure, enabling simultaneous interaction with multiple molecular targets.^[^ ^1^ ^]^ This multitarget interaction principle is closely related to the concept of polypharmacology, which forms the theoretical foundation of multitarget drug design and development. Polypharmacology refers to the ability of a single drug to interact with multiple biological targets, offering potential advantages in treating complex diseases by modulating several pathways simultaneously. This approach contrasts with the classical ‘one target‐one drug’ paradigm and has gained increasing attention in recent years.^[^ ^2^ ^]^

The ‘one target‐one drug’ model has been the dominant strategy in drug discovery, based on the assumption that disease symptoms or progression can be mitigated by precisely activating or inhibiting a single biological target.^[^ ^2b^ ^,^ ^3^ ^]^ However, drugs designed with this approach have proven insufficient for addressing complex and multifactorial neurodegenerative disorders, such as Alzheimer's, Parkinson's, and amyotrophic lateral sclerosis (ALS). These drugs have also been inadequate for treating diseases caused by microorganisms that develop drug resistance, including cancer, malaria, leishmaniasis, and COVID‐19.^[^ ^3^ ^,^ ^4^ ^]^ Recent studies suggest that regulating multiple targets concurrently may result in higher therapeutic efficacy and safety compared to single‐target drugs.^[^ ^5^ ^]^

**Figure 1:**
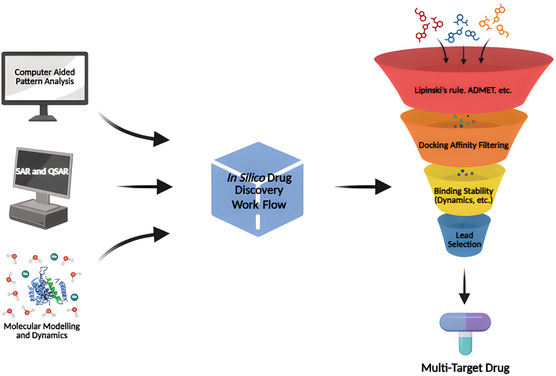
Design and development of multitarget drugs. [This figure was created with BioRender.com (https://www.biorender.com/)].

Multitarget drugs offer several advantages, including a reduced risk of drug–drug interactions, broader efficacy in certain clinical settings, enhanced patient convenience, and decreased treatment complexity.^[^ ^6^ ^]^ The design of multitarget drugs employs techniques including molecular docking, structure–activity relationship studies (SAR), quantitative SAR (QSAR) studies, virtual screening, and pharmacophore combination method (**Figure** **1**).^[^ ^7^ ^]^

In support of the growing interest in multitarget strategies, recent drug approval trends also reflect a shift toward polypharmacology. A review of pharmaceutical approvals by the European Medicines Agency (EMA) and drugs marketed in Germany between 2023 and 2024 identified 18 out of 73 newly introduced drugs as aligning with the principles of polypharmacology. These include ten antitumor agents (Loncastuximab tesirine, Epcoritamab, Glofitamab, Elranatamab, and so on), five drugs for autoimmune/inflammatory diseases (Ritlecitinib Etrasimod Zilucoplan Elafibranor Sparsentan), one antidiabetic agent (Tirzepatide) with antiobesity effects, one modified corticosteroid (Vamorolone), and one drug for hand eczema (Delgocitinib). These agents exemplify the increasing adoption of multitarget or polypharmacological approaches in contemporary clinical pharmacotherapy.^[^ ^8^ ^]^

In the synthesis of multitarget drugs, reactions such as copper‐catalyzed azide‐alkyne cycloaddition (CuAAC), the Stille reaction, the Heck reaction, the Suzuki cross‐coupling reaction, the Sonogashira reaction, and the Diels–Alder reaction are commonly employed due to their high efficiency and versatility.^[^ ^4d^ ^]^

The literature reviewed in this study encompasses research conducted between 2010 and 2024. A comprehensive literature search was performed using international databases, such as PubMed and Web of Science, employing keywords such as ‘multitarget drugs, Alzheimer, Parkinson, cancer, molecular docking, synthesis reactions’, and various combinations thereof. The screening process was completed in Dec. 2024.

Previous reviews^[^ ^9^ ^]^ have predominantly focused on specific health conditions; however, this study explores the synthetic reactions and applications employed in the development of multitargeted drugs for diseases with complex etiologies, including neurodegenerative disorders, cancer, and certain microbial infections (such as malaria, leishmaniasis, and COVID‐19), which have developed resistance to existing treatments. In this context, the urgent need for novel therapeutic approaches and drugs with diverse mechanisms of action is highlighted.

## 2 Multitargeted Drug Definition

Multitargeted drugs, which are skeletons that can interact with two or more molecular targets simultaneously, contain pharmacophore groups of two or more drugs with similar or different mechanisms‐of‐action in a single molecule.^[^ ^10^ ^]^ Researchers have developed such molecules to address multiple disease‐related targets, thereby improving treatment outcomes.^[^ ^11^ ^]^ Ligands formulated for this purpose are described using terms, such as binary, two‐component, bivalent, dimeric, ternary, mixed, or balanced, often accompanied by suffixes, such as ligand, inhibitor, agonist, antagonist, conjugate, or blocker. To standardize nomenclature and facilitate scientific discussion, researchers have recommended the adoption of the term ‘designed multiple ligands’ for these compounds.^[^ ^11^ ^,^ ^12^ ^]^

## 3 We Need Multitarget Drugs—but why?

The ‘lock and key’ model proposed by Erlich over a century ago formed the basis for the ‘one target, one drug’ hypothesis. In recent decades, therapeutic drugs have been developed to selectively modulate a single target, aiming to minimize undesirable effects.^[^ ^13^ ^]^ However, drugs designed under this paradigm often fail to provide adequate therapeutic effects for complex diseases, including neurodegenerative disorders (e.g., Alzheimer's disease (AD), Parkinson's disease, schizophrenia), mood disorders, chronic inflammation, and cancer.^[^ ^13b^ ^,^ ^14^ ^]^ The multifactorial nature of these diseases necessitates the development of drugs that interact with multiple targets, as single‐target approaches frequently prove inadequate for effective treatment.^[^ ^15^ ^]^

Multitarget drugs exemplify polypharmacology, offering the advantage of simultaneous modulation of multiple targets and pathways involved in disease progression. This ability reduces the likelihood of resistance development and enhances therapeutic outcomes by addressing disease heterogeneity. Incorporating polypharmacology into drug design challenges traditional paradigms and requires innovative approaches in both computational and synthetic chemistry.^[^ ^2^ ^]^

However, the multitarget drug development strategy presents a range of challenges and risks.^[^ ^16^ ^]^ Drug promiscuity, which refers to the nonselective interaction of a compound with multiple biological targets, can increase the risk of toxicity and adverse effects, potentially outweighing therapeutic benefits.^[^ ^17^ ^]^ Similarly, off‐target interactions may lead to unintended pharmacological responses, thereby elevating the likelihood of clinical failure.^[^ ^2b^ ^]^ Moreover, it is often unclear whether multitarget activity arises from rational drug design or occurs merely by chance, introducing the notion of ‘chance polypharmacology.’ As such, despite the potential advantages of polypharmacological agents, the design and evaluation of multitarget drugs demand a cautious and critical approach.^[^ ^4b^ ^]^

Among these challenges, one of the primary concerns is drug resistance, which significantly hampers the treatment of conditions, such as cancer, epilepsy, malaria, and bacterial infections.^[^ ^18^ ^]^ The increasing resistance of bacteria to antibiotics, coupled with the decreasing rate of novel antibiotic discovery, has led to a global health crisis.^[^ ^19^ ^]^ Despite extensive research efforts, the rate of new antibacterial drug discovery has not kept pace with the rapid emergence of resistance, posing a significant concern.^[^ ^20^ ^]^ Pathogens acquire resistance through various mechanisms, including alternative metabolic pathways, modifications in drug targets, enzymatic inactivation of pharmaceuticals, active drug efflux, and reduced membrane permeability.^[^ ^21^ ^]^ Drug resistance frequently arises due to the modulation of a single protein or pathway by a drug, thereby diminishing its efficacy over time.^[^ ^22^ ^]^ The demand for antibiotics with both broad‐spectrum activity and efficacy against drug‐resistant strains has increased, yet the conventional ‘single target, single molecule’ paradigm has failed to meet this need.^[^ ^23^ ^]^ This resistance has highlighted the limitations of single‐target drugs, which often fail to produce the expected therapeutic outcomes due to bacterial mutations and intrinsic resistance mechanisms.^[^ ^18b^ ^]^ Unlike single‐target drugs, multitarget drugs are less susceptible to resistance arising from single‐point mutations, making them a promising alternative for combating resistant microorganisms.^[^ ^24^ ^]^

As a result, single‐target pharmaceuticals have proven inadequate for addressing multigenic disorders, such as AD^[^ ^25^ ^]^ and cancer,^[^ ^26^ ^]^ as well as intricate conditions affecting multiple cell types and tissues, such as diabetes.^[^ ^27^ ^]^ The effectiveness of single‐target drugs in treating epilepsy, cancer, and microbial infections often declines due to the emergence of drug resistance. This challenge has underscored the urgent need for drugs capable of simultaneously modulating multiple targets, thereby enhancing therapeutic efficacy and reducing the likelihood of resistance development.

## 4 Drug Repurposing

Drug repurposing refers to the identification of additional therapeutic applications for drugs that are in the experimental stage, currently in use, discontinued, or rendered obsolete due to factors, such as drug resistance or adverse effects.^[^ ^28^ ^]^

**Figure 2:**
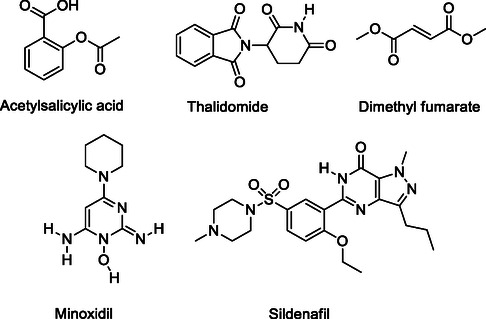
Some repurposing marketed drugs.

The development and introduction of a new pharmaceutical agent through research and development (R&D) is a time‐consuming and financially demanding process.^[^ ^29^ ^]^ Identifying an active ingredient and subsequently developing it into a viable pharmaceutical compound typically takes eight to ten years.^[^ ^30^ ^]^ Initial research begins with a pool of 100,000 synthesized molecules, ultimately yielding only two to four candidates that may be classified as novel pharmaceuticals, ready for market introduction. The likelihood of a newly developed drug failing during clinical trials is substantial, with half of the molecules not succeeding in phase 3, representing a significant misallocation of financial resources.^[^ ^31^ ^]^ In essence, identifying a novel active ingredient and developing it into a dependable pharmaceutical with established indications is an expensive and complex endeavor requiring considerable time investment. In contrast, drug repurposing can occur more safely, rapidly, and cost‐effectively.^[^ ^32^ ^]^ Aspirin, thalidomide, sildenafil, minoxidil, and dimethyl fumarate (DMF) are prominent examples of repurposing drugs^[^ ^31^ ^,^ ^33^ ^]^ as illustrated in **Figure** **2**.

Thalidomide, a glutamic acid derivative and glutethimide analog with the chemical name 2‐(2,6‐dioxo‐3‐piperidyl)isoindole‐1,3‐dione,^[^ ^34^ ^]^ is a drug with antiemetic and hypnotic properties that was first synthesized in Germany in 1954 and marketed in 1956.^[^ ^35^ ^]^ Due to its antiemetic effects, thalidomide was widely used to manage nausea and vomiting in pregnant women.^[^ ^36^ ^]^ However, following its introduction in Australia, Japan, and European countries, birth defects, including phocomelia (limb deformities), were reported in ≈12,000 newborns, prompting the drug's withdrawal from the market in 1961.^[^ ^37^ ^]^ In 1964, evidence emerged suggesting that thalidomide was effective in treating erythema nodosum leprosum, which was attributed to its inhibition of the proinflammatory cytokine tumor necrosis factor‐alpha (TNF‐α).^[^ ^38^ ^]^ Consequently, Celgene repurposing thalidomide in 1998 for the treatment of leprosy‐related conditions.^[^ ^33^ ^]^ Further investigation into the teratogenic mechanisms of thalidomide revealed its antiangiogenic properties, demonstrating its ability to inhibit tumor hypervascularity, growth, and metastasis.^[^ ^37^ ^]^ Given its demonstrated efficacy, thalidomide was repurposed in 2006 for the treatment of multiple myeloma.^[^ ^39^ ^]^

Dimethyl fumarate, scientifically known as dimethyl (2*E*)‐but‐2‐enedioate (DMF), is the methyl ester of fumaric acid, a compound named after the soil‐dwelling plant *Fumaria officinalis*.^[^ ^40^ ^]^ The first medical application of DMF dates back to 1959, when German chemist Schweckendiek investigated the use of fumaric acid esters for the treatment of psoriasis—a chronic skin condition that currently affects approximately 2%–3% of the global population across nearly all age groups.^[^ ^41^ ^]^ A formulation combining DMF with three ethyl hydrogen fumarate salts was later developed and became the first licensed drug for psoriasis treatment, approved in 1994 by the German Medicines Agency under the trade name Fumaderm.^[^ ^42^ ^]^ The clinical use of DMF broadened in the early 2000s through the ‘drug repurposing’ approach.^[^ ^43^ ^]^ Subsequent studies revealed that DMF exerts neuroprotective and antioxidant effects via the activation of the nuclear factor erythroid 2‐related factor 2 (Nrf2) pathway.^[^ ^44^ ^]^ Based on these findings, the United States Food and Drug Administration (FDA) approved DMF in 2013 for the treatment of multiple sclerosis (MS) under the brand name Tecfidera.^[^ ^45^ ^]^

Minoxidil, chemically designated as 6‐(1‐piperidinyl)‐2,4‐pyrimidinediamine 3‐oxide, contains a piperidine moiety attached to a pyrimidine ring in its structure. It was introduced in the 1970s under the brand name Loniten for the treatment of hypertension.^[^ ^46^ ^]^ By activating adenosine triphosphate‐sensitive potassium channels, it induces arterial dilation and lowers blood pressure, thus classifying it as a potent vasodilator.^[^ ^47^ ^]^ In the following years, increased hair growth on the scalp and face was observed as a side effect, drawing the attention of researchers. This effect was subsequently investigated through dermatological studies, leading to the development of a topical formulation of minoxidil.^[^ ^48^ ^]^ Consequently, in the 1980s, it received FDA approval for the treatment of hair loss under the brand name Rogaine.^[^ ^49^ ^]^

Sildenafil is a phosphodiesterase type 5 inhibitor that was synthesized by Pfizer in 1989. It facilitates the relaxation of smooth vascular muscles by inhibiting the degradation of cyclic guanosine monophosphate. Although it was initially developed for the treatment of angina (chest pain) and pulmonary arterial hypertension, clinical phase studies also revealed its effectiveness in treating erectile dysfunction. Based on this new indication, it was launched in 1998 under the brand name Viagra, becoming a successful example of drug repurposing.^[^ ^50^ ^]^

Aspirin, whose active ingredient is acetylsalicylic acid, and which originates from the willow tree, was first introduced by Bayer in 1899.^[^ ^51^ ^]^ It exerts its anti‐inflammatory effects by inhibiting cyclooxygenase (COX) enzymes, which are involved in the synthesis of prostaglandins responsible for inflammation.^[^ ^52^ ^]^ Recent studies have shown that aspirin is also effective in both the prevention and treatment of several cancers, such as esophageal, gastric, colorectal, prostate, and breast cancer.^[^ ^53^ ^]^

## 5 Benefits of Multitarget Drugs

Many diseases—including neurodegenerative disorders, mood disturbances, and cancer—exhibit complex pathophysiologies that cannot be effectively managed by drugs targeting a single molecular entity.^[^ ^54^ ^]^

The dysregulation of multiple signaling pathways and various physiological processes are recognized as a key contributor to neurodegeneration,^[^ ^55^ ^]^ cancer initiation, metastasis, and cellular proliferation.^[^ ^56^ ^]^ To address diseases arising from such multifactorial disruptions, it is essential to modulate several targets simultaneously, thereby restoring physiological homeostasis. This goal may be achieved either by administering multiple drugs concurrently—a strategy known as polypharmacy—or by employing a single drug capable of interacting with several targets at once.^[^ ^57^ ^]^

However, polypharmacy, defined as the simultaneous use of multiple medications, increases the risk of drug–drug interactions.^[^ ^58^ ^]^ Moreover, it presents several drawbacks, including elevated treatment costs due to the need for multiple drugs for a single condition, a heightened risk of adverse effects from off‐target interactions, and the possibility that one drug may reduce the bioavailability or solubility of another, thereby increasing toxicity.^[^ ^59^ ^]^ In this context, drugs with multitarget activity can potentially replace some of the medications used in combination therapy, significantly reducing the reliance on polypharmacy.^[^ ^60^ ^]^ Reducing the number of drugs in a treatment regimen simplifies the pharmacokinetic landscape, decreases the incidence of side effects and interactions, and enhances patient adherence.^[^ ^61^ ^]^ Furthermore, engaging multiple targets may produce synergistic or additive effects that enhance therapeutic efficacy.^[^ ^62^ ^]^ Additionally, developing multitarget drugs typically requires fewer clinical trials, offering substantial benefits in terms of both financial expenditure and development timelines.^[^ ^4c^ ^]^

## 6 Pharmacophore Groups

The International Union of Pure and Applied Chemistry (IUPAC) defines a pharmacophore as the ensemble of steric and electronic features necessary to ensure optimal intermolecular interactions with a specific biological target, thereby triggering or inhibiting a biological response. Alternatively, a pharmacophore may be understood as the common structural or functional elements present in a series of compounds that are essential for activating a particular biological target.^[^ ^63^ ^]^ Significant heterocyclic structures, including pyrrolidine,^[^ ^64^ ^]^ thiazole,^[^ ^65^ ^]^ triazole,^[^ ^66^ ^]^ indole,^[^ ^67^ ^]^ imidazole,^[^ ^68^ ^]^ coumarin,^[^ ^69^ ^]^ chromone,^[^ ^70^ ^]^ benzenesulfonamide,^[^ ^71^ ^]^ benzodiazepine,^[^ ^72^ ^]^ and thiourea,^[^ ^73^ ^]^ are recognized as important pharmacophores due to their diverse pharmacological activities, playing a crucial role in drug research and development.^[^ ^74^ ^]^ The following discussion focuses on molecules that incorporate specific pharmacophores and exhibit multiple pharmacological effects.

Chromone is a heterocyclic compound chemically known as 4*H*‐1‐benzopyran‐4‐one and its derivatives display a range of pharmacological activities, including antihistaminic, anti‐inflammatory, antidiabetic, antitumor, antimicrobial, antifungal effects, as well as immune system stimulation—according to several studies.^[^ ^75^ ^]^ The activity of novel chromone based compounds has been investigated against key enzymes, such as acetylcholinesterase (AChE), β‐secretase‐1 (BACE‐1), and monoamine oxidase B (MAO‐B).^[^ ^76^ ^]^

Liu et al. designed and synthesized a series of chromone‐2‐carboxamido‐alkylbenzylamine derivatives capable of interacting with multiple targets for the treatment of AD. The synthesized compound **1** exhibited significant inhibitory activity against AChE (Rat AChE IC_50_: 0.07 µM, Ee AChE IC_50_: 0.55 µM), moderate antioxidative properties [oxygen radical absorbance capacity fluorescein (ORAC‐FL) value: 0.83 eq. (trolox equiv)], selective biometal chelating capabilities, and inhibitory effects on both self‐induced and copper ion (Cu^+2^)‐induced amyloid‐β (Aβ) aggregation (Self‐induced Aβ_1–42_ aggregation: 59.2% inhibition at 25 µM, Cu^+2^ induced Aβ_1–42_ aggregation: 48.3% inhibition at 25 µM).^[^ ^77^ ^]^


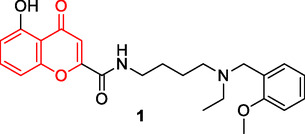


This research indicates that compounds with substituted chromone rings can serve as pharmacophore groups in multitarget drug development.

Benzenesulfonamides exhibit a broad range of pharmacological activities, including antibacterial, anticancer, carbonic anhydrase (CA), and cholinesterase inhibitory effects, as demonstrated in several studies.^[^ ^71b^ ^,^ ^78^ ^]^ This diverse activity profile emphazises the potential of the benzenesulfonamide scaffold as a valuable pharmacophore in multitarget drug design. Notably, benzenesulfonamides are classical zinc‐binding groups in CA inhibitors;^[^ ^79^ ^]^ the sulfonamide moiety coordinates with the catalytic Zn^2^^+^ ion and forms hydrogen bonds with key residues within the enzyme's binding pocket.^[^ ^80^ ^]^

Ragab et al. designed and synthesized a series of compounds based on 4‐(5‐amino‐pyrazol‐1‐yl)benzenesulfonamide, demonstrating significant inhibition of cyclooxygenase‐2 (COX‐2), 5‐lipoxygenase (5‐LOX), and CA, thereby indicating their potential as effective anti‐inflammatory agents. Specifically, compounds with –Ph, hydroxyphenyl, and thiophenyl substituents, respectively, **2** (IC_50_ COX‐2: 49 nM, 5‐LOX: 2.4 nM), **3** (IC_50_ COX‐2: 60 nM, 5‐LOX: 1.9 nM), and **4** (IC_50_ COX‐2: 60 nM, 5‐LOX: 2.5 nM) exhibited significant inhibitory activities against COX‐1, COX‐2, and 5‐LOX.^[^ ^81^ ^]^


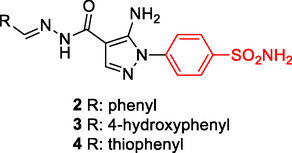


The indole ring, a flexible pharmacophore, exhibits diverse biological activities by interacting with multiple receptors.^[^ ^82^ ^]^ Indole scaffolds engage in π–π stacking and hydrogen bonding due to the NH group and aromatic ring. The conjugated aromatic structure of indole enables diverse interactions with biological macromolecules, including hydrogen bonding, π–π stacking, and hydrophobic interactions, which are crucial for achieving strong binding affinity and selectivity toward therapeutic targets.^[^ ^83^ ^]^ Its antimicrobial activity,^[^ ^67b^ ^]^ α‐amylase^[^ ^84^ ^]^ and monooxime inhibitory effects, antimalarial properties,^[^ ^85^ ^]^ and anticancer^[^ ^86^ ^]^ potential have been extensively documented.

Nerella et al. designed and synthesized a series of multifunctional hybrid compounds by conjugation of indole and deoxyvaccinone pharmacophore groups. Compound **5** was found to inhibit both AChE (IC_50_: 0.12 µM) and butyrylcholinesterase (BuChE) (IC_50_: 0.15 µM). Furthermore, compound **5** was observed to inhibit both self‐induced Aβ_1‐42_ (IC_50_: 1.21 µM) and AChE‐induced Aβ_1‐42_ aggregation.^[^ ^87^ ^]^


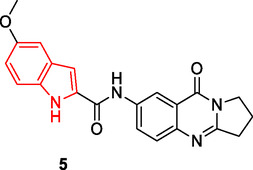


The triazole heterocyclic ring represents a key pharmacophore in the development of multitarget drugs. Triazoles, also known as pyrroliazoles, are five‐membered unsaturated heterocyclic rings containing three nitrogen atoms.^[^ ^88^ ^]^ Numerous studies have highlighted their broad pharmacological potential, including antituberculosis,^[^ ^89^ ^]^ anticonvulsant,^[^ ^90^ ^]^ antifungal,^[^ ^91^ ^]^ analgesic,^[^ ^92^ ^]^ and antiprotozoal^[^ ^93^ ^]^ activities. Their unique structural features enable triazole derivatives to interact readily with a wide range of enzymes and receptors in biological systems through various weak interactions, such as coordination bonds, hydrogen bonds, ion–dipole interactions, cation–π and π–π stacking, hydrophobic effects, and van der Waals forces.^[^ ^94^ ^]^

Hydroxy‐1,2,3‐triazole derivatives **6**, **7** have shown promising biological activities, including antileishmanial effects,^[^ ^95^ ^]^ activity against the F508del cystic fibrosis transmembrane conductance regulator (CFTR) mutation (the most common cause of cystic fibrosis),^[^ ^96^ ^]^ and antiviral properties against Zika and Chikungunya viruses.^[^ ^97^ ^]^ Compounds **6a**, **6b**, and **7** exhibited synergistic effects in combination with clinically approved drug VX‐661 or the treatment of cystic fibrosis. Among them, **6b** displayed potent antileishmanial activity, with IC_50_ values of 15.52 ± 3.78 μM against promastigotes and 4.10 ± 1.14 μM against intracellular amastigotes, along with approximately 20‐fold selectivity based on its 50% cytotoxic concentration (84 μM) in BALB/c rabbit peritoneal macrophages. In antiviral assays, **6c** was the most effective inhibitor of Zika virus, showing over 90% inhibition at 20 μM, while **6d** exhibited the highest inhibition of Chikungunya virus replication (>90%).


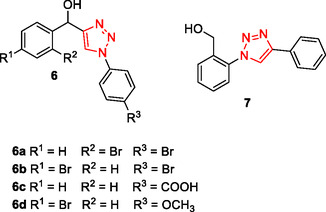


Kaur et al. designed and synthesized a series of triazole‐based compounds as multitarget ligands for the treatment of AD. Compound **8** was found to possess the ability to decompose preexisting Aβ_42_ aggregates (Inhibition of Aß_42_ aggregation: 96.89%, dissaggregation of Aß_42_ fibrils: 81.89%, IC_50_ for Aß_42_ aggregation inhibition: 8.065 ± 0.129 µM) exhibit metal chelating characteristics, and impede the aggregation of Aβ_42_ induced by Cu^+2^ (inhibition of Cu^+2^‐Aß_42_ aggregation: 65.49%, dissaggregation of Cu^+2^‐Aß_42_ fibrils: 61.42%) Furthermore, it was observed that compound **8** effectively regulated the generation of reactive oxygen species (ROS) by inhibiting the copper redox cycle.^[^ ^98^ ^]^


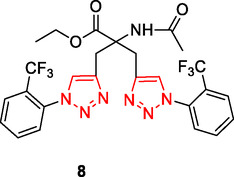


## 7 Methods used in Multitargeted Drug Design

### 7.1 Molecular Docking

Molecular docking is a computational technique used to simulate interactions between a molecule and a target protein at atomic level. It helps determine the optimal conformation of a ligand within a protein's binding site and predicts the stability of ligand–protein complexes. This approach is crucial for identifying potential drug candidates and understanding ligand–receptor interactions.^[^ ^99^ ^]^ Molecular docking also provides insights into ligand binding patterns, compares binding energies among different compounds, and estimates the stability of ligand–target complexes.^[^ ^100^ ^]^ AutoDock, GOLD, and CDOCKER are the most favored docking software applications.^[^ ^100^ ^,^ ^101^ ^]^

Molecular docking plays a pivotal role in assessing the binding affinity of chemical compounds to their target receptors, evaluating drug selectivity, and predicting the potential interactions of multiple drugs with a single target.^[^ ^102^ ^]^ This method is particularly valuable for designing multitarget drugs by analyzing how existing pharmaceuticals interact with different receptors.

Ali et al. employed molecular docking to screen multitarget compounds and assess their protective effects against acetaminophen‐induced liver damage. Using a library of 40 compounds derived from *Curcuma longa* and *Cinnamomum seylanicum*, they identified quercetin and curcumin as the most promising hepatoprotective agents. These compounds demonstrated strong binding affinities to cytochrome P4502E1 (CYP2E1), mitogen‐activated protein kinase, and Toll‐like receptor 4, all of which are implicated in liver damage. Furthermore, quercetin and curcumin reduced the viability of hepatocellular carcinoma (HepG2 and Huh7) cell lines, suggesting potential anticancer properties.^[^ ^103^ ^]^

### 7.2 SAR and QSAR

SAR analysis is a modeling approach that explores both qualitative and quantitative correlations between chemical structures and their biological activities.^[^ ^104^ ^]^ SAR studies help identify pharmacophore groups responsible for a compound's biological activity while also determining structural features that may contribute to adverse effects. This knowledge facilitates the development of novel drugs with improved efficacy, reduced toxicity, and distinct mechanisms of action compared to existing treatments.^[^ ^105^ ^]^

Using multidimensional SAR analysis, Tassini et al. identified a series of compounds with phosphatidylinositol 4‐kinase IIIβ (PI4KIIIβ) inhibitory properties for the treatment of cystic fibrosis. These compounds were found to correct misfolded CFTR proteins carrying the Phe 508 deletion. Additionally, they exhibited synergistic activity with the class I corrector lumacaftor (VX‐809). Compound **9** displayed strong inhibition of PI4KIIIβ (IC_50_: 2.10 µM) and also demonstrated antiviral activity against specific picornaviruses.^[^ ^106^ ^]^


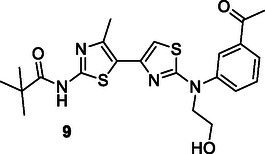


QSAR analysis seeks to establish statistically significant correlations between molecular structures and their biological activities through computational modeling. This approach enables researchers to predict the biological activity of novel compounds, determine key structural features required for activity, and optimize existing drug candidates.^[^ ^107^ ^]^ Given the vast number of possible chemical substitutions, QSAR provides a rational strategy for selecting optimal molecular modifications without synthesizing an exhaustive range of compounds.^[^ ^105^ ^]^

**Figure 3:**
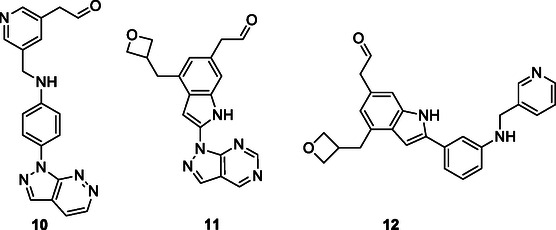
Proposed molecules as potential anticolorectal cancer.

Speck‐Planche et al. applied QSAR modeling to analyze a diverse set of biologically active compounds and identify pharmacophore groups responsible for anticolorectal cancer activity. Compounds **10–12**, exhibiting multifaceted anticolorectal cancer activity, were obtained by integrating molecules identified as having such activity by the QSAR technique (**Figure** **3**).^[^ ^108^ ^]^ This study demonstrates that the biological activities of compounds, suitable as pharmacophore groups, may be assessed using QSAR, facilitating their application in multitarget drug development.

### 7.3 Virtual Screening

Virtual screening is an in silico technique used to identify potential bioactive molecules by assessing the predicted affinities and possible binding configurations of drug candidates, which are small molecules that may serve as drugs or pharmacophores of the lead compound when interacting with the target protein.^[^ ^109^ ^]^ The virtual screening method facilitates the rapid and cost‐effective design of lead compounds and drug improvements in new drug development.^[^ ^110^ ^]^ It aids in designing novel bioactive compounds by identifying the most suitable chemicals that can interact with the target of interest from a structural database.^[^ ^110a^ ^]^

Knox et al. employed a virtual screening strategy to identify two targeted compounds with inhibitory activity against heat shock protein 90 (HSP 90) and tubulin. By examining a structural database of ≈160,000 compounds, they identified compound **13**, which exhibits inhibitory activity against both HSP90 and tubulin.^[^ ^111^ ^]^


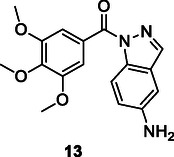


**Figure 4:**
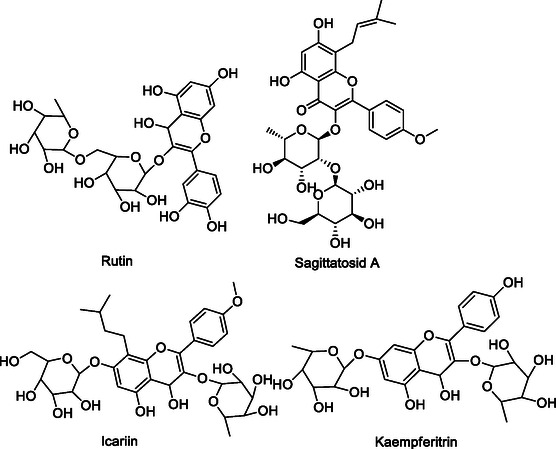
Cathepsin K, V‐ATPase, and αvβ3 targets herbal compounds.

Chaichit et al. conducted further research using the virtual screening technique to explore molecular interactions with cathepsin K, V‐ATPase, and αvβ3 integrin, which are targets of interest in osteoporosis. Their virtual screening of 108 herbal compounds revealed that rutin, sagittatoside A, icariin, and kaempferitrin exhibited significant binding activity against these targets. This suggests that these compounds may serve as pharmacological agents that influence multiple targets in the context of osteoporosis or within the framework of multitarget drug design (**Figure** **4**).^[^ ^112^ ^]^ These studies, and similar ones, demonstrate that virtual screening plays a critical role in the design and development of multitarget drugs.

### 7.4 Computer‐Aided Pattern Analysis (C@PA)

C@PA is a data‐driven computational methodology specifically developed for multitarget drug discovery. Unlike traditional approaches, such as pharmacophore modeling, docking, or QSAR, CAPA does not rely on predefined pharmacophoric features. Instead, it identifies bioactive compounds based on the analysis of chemical patterns and biological activity profiles. This approach has demonstrated superior predictive power for multitarget activities and has been successfully applied in recent multitarget drug development studies. C@PA, also known as computer‐aided pattern scoring (C@PS), involves the extraction of multitarget fragments and subsequent multitarget fingerprints from compounds functionally evaluated against two or more targets, and the application of these fragments and fingerprints across chemical space to predict potential polypharmacological ligands.^[^ ^2b^ ^,^ ^113^ ^]^

Focusing on ATP‐binding cassette transporter (ABCA1), a challenging pharmacological target involved in malignant, metabolic, and neurodegenerative diseases, Stephan et al. developed a novel chemoinformatics workflow called C@PS to identify new ligands. Using a unique dataset containing critical information on ABCA1, they achieved a 95.5% accuracy rate in identifying compounds with high potency and structural diversity. These findings offer a valuable example not only for the deorphanization of ABCA1 but also for broader multitarget drug discovery efforts targeting pharmacologically orphan proteins.^[^ ^113c^ ^]^

## 8 Pharmacophore Combination Approach

**Figure 5:**
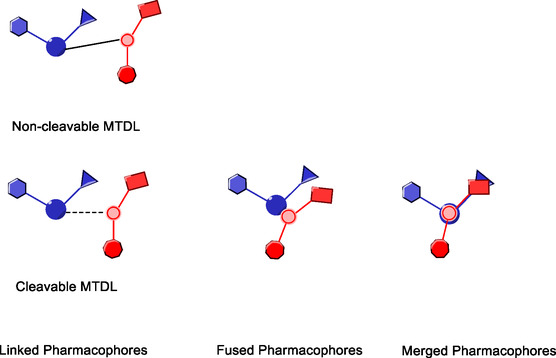
Linked, fused, and merged pharmacophores.^[^ ^4b,d^ ^]^

The integration of pharmacophores derived from selective compounds is a widely used approach in multitarget drug design.^[^ ^114^ ^]^ This strategy allows the combination of pharmacophore groups from selective compounds into a single molecule, thereby exhibiting the biological activities of these compounds to affect multiple targets.^[^ ^115^ ^]^ Multitarget directed ligands (MTDL) can be designed by linking pharmacophore groups using either cleavable or noncleavable linkers, or by superimposing them based on the structural similarities of the pharmacophores.^[^ ^4d^ ^]^ MTDLs developed using this approach are categorized into three groups: linked (or conjugates), fused, and merged species, depending on the extent of overlap of their pharmacophores (**Figure** **5**).^[^ ^116^ ^]^

### 8.1 Linked Pharmacophores

MTDLs in this category are formed by linking molecular structures containing pharmacophore groups that interact with the target independently through distinct linkers, which are not part of the selective ligands.^[^ ^116^ ^]^ Optimizing the linker length, position, and structure is essential to maintain the activity of the original compound.^[^ ^117^ ^]^ Linked MTDLs are further categorized into two classes: cleavable and noncleavable, based on the characteristics of the linker group.^[^ ^7b^ ^]^ Cleavable MTDLs utilize metabolizable linkers, such as esters, to enable the release of two ligands capable of interacting with each target independently.^[^ ^7b^ ^,^ ^116^ ^]^ Noncleavable conjugates are constructed using metabolically stable linkers.^[^ ^116^ ^]^

**Figure 6:**
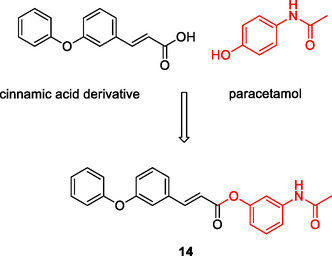
Molecular structure of cleavable MTDL obtained by binding paracetamol and cinnamic acid via the ester group.

Peperidou et al. developed a multitarget compound demonstrating LOX enzyme inhibition and analgesic properties by combining cinnamic acid and paracetamol pharmacophores through an ester group.^[^ ^118^ ^]^ Hydrolysis of the ester group results in the release of paracetamol and cinnamic acid pharmacophores, which bind to target receptors independently. The designed compound **14** (LOX IC_50_: 0.34 µM Analgesic activity (0.01 mmol/0.1 kg): 98.1%) exemplifies a cleavable MTDL (**Figure** **6**).

**Figure 7:**
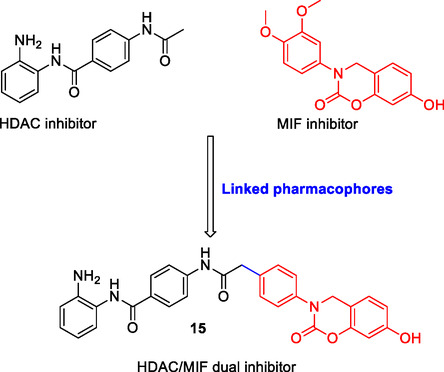
Designed MTDL **15** as HDAC/MIF dual inhibitor by the linked pharmacophore approach.

Cao et al. designed and synthesized a multitarget molecule **15** (**Figure** **7**) by linking the pharmacophore groups of compounds that inhibit histone deacetylase (HDAC) and macrophage migration inhibitory factor (MIF). The synthesized compound was found to retain inhibition properties for both HDAC and MIF.^[^ ^119^ ^]^

### 8.2 Fused Pharmacophores

MTDLs created by arranging pharmacophore groups in a configuration lack pharmacophore overlap and identifiable linker groups.^[^ ^120^ ^]^

**Figure 8:**
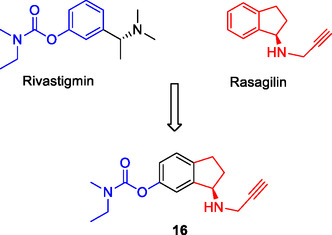
MTDL **16** is designed by combining pharmacophore groups.

Sterling et al. designed compound **16** (**Figure** **8**), which integrates the phenyl group from rivastigmine and the dihydroindene group from rasagiline into a fused structure, demonstrating efficacy against both AChE (IC_50_: 52.4 µM), and monoamine oxidase A (MAO‐A) (IC_50_: 85 µM) and MAO‐B (IC_50_: 120 µM) enzymes.^[^ ^121^ ^]^ The absence of a linker group or pharmacophore overlaps in the structure of this compound exemplifies an MTDL resulting from the fusion of pharmacophores.

**Figure 9:**
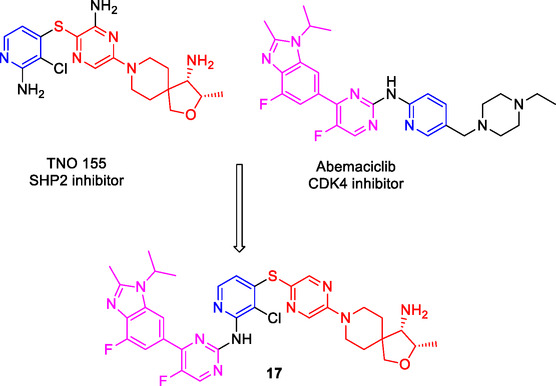
MTDL **17** as SHP2 and CDK4 inhibitor.

Chen et al. designed and synthesized a series of compounds that inhibit protein tyrosine phosphatase‐2 (SHP2) and cyclin dependent kinase 4 (CDK4) by utilizing the fused pharmacophore strategy. Among the compounds, compound **17** (**Figure** **9**) exhibited the most potent inhibitory activities for SHP2 (IC_50_: 4.3 nM) and CDK4 (18.2 nM).^[^ ^122^ ^]^

### 8.3 Merged Pharmacophores

In merged pharmacophores, the pharmacophore groups are synthesized by identifying the relevant components of each receptor and employing the shared characteristics in the structure of lead ligands to develop MTDLs with a high degree of overlapping pharmacophore groups. These integrated pharmacophores are designed to achieve structures with enhanced physicochemical properties, reduced molecular weights, and simplified configurations.^[^ ^4d^ ^]^

**Figure 10:**
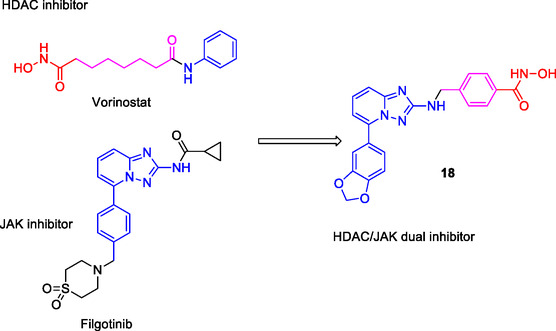
MTDL **18** by merging pharmacophores.

Xu et al. designed triazolopyridine derivatives as dual HDAC/JAK inhibitors by merging the pharmacophores of the HDAC inhibitor vorinostat and the JAK inhibitor filgotinib into a single molecule. Compound **18** (**Figure** **10**) emerged as a dual HDAC/JAK inhibitor, exhibiting the highest cytotoxicity against MDA‐MB‐231 and RPMI‐8226 cancer cell lines. It demonstrated IC_50_ values of 25.4 nM for HDAC1, 4.3 nM for HDAC6, 165 nM for JAK1, and 178 nM for Janus kinase 2 (JAK2).^[^ ^123^ ^]^

**Figure 11:**
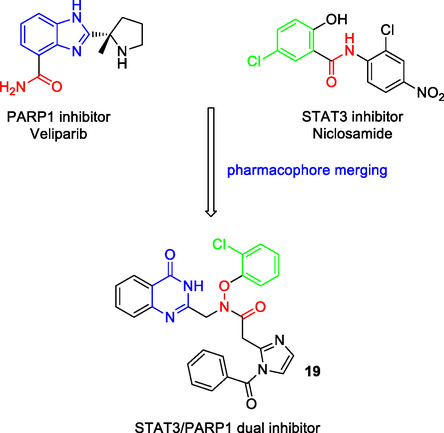
MTDL **19** as PARP1 and STAT3 inhibitor.

Dilipkumar et al. synthesized 30 quinazolinone derivatives that are dual inhibitors of poly (ADP‐ribose) polymerase (PARP1) and signal transducer and activator of transcription 3 (STAT3) by using the merged pharmacophore strategy. The synthesized compounds were evaluated for their dual inhibitory effects on PARP1 and STAT3 through docking, MM‐GBSA, and molecular dynamics simulation studies. Compound **19** (**Figure** **11**) was identified as the most effective in molecular dynamics simulations and MM‐GBSA calculations.^[^ ^124^ ^]^

## 9 Some Synthesis Methods of Multitargeted Drugs

The following synthetic methods are frequently employed in the development of multitargeted drugs, not only for their efficiency and modularity but also for their ability to generate pharmacophore‐rich scaffolds with activity across different therapeutic areas. Below, each method is illustrated with selected examples that demonstrate the structural rationale behind their biological activity.

### 9.1 CuAAC Reaction

**Scheme 1:**
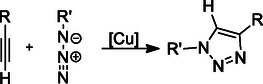
CuAAC reaction procedure.

The CuAAC ‘click’ reaction is widely utilized in medicinal chemistry for constructing 1,2,3‐triazole rings, which serve as stable, bioisosteric linkers with intrinsic pharmacological activity. It involves the reaction of azides with terminal alkynes in the presence of a copper catalyst to selectively yield 1,4‐disubstituted 1,2,3‐triazoles (**Scheme** **1**).^[^ ^125^ ^]^ This reaction proceeds efficiently at ambient temperature, typically affording products in up to 95% yield with minimal byproducts. The chemical inertness of the triazole ring, combined with the simplicity and reliability of the CuAAC process, makes it an attractive strategy for linking diverse pharmacophores.^[^ ^4d^ ^]^

Quinazoline–1,2,3‐triazole hybrids, synthesized via the CuAAC reaction, were developed as multiple inhibitors targeting epidermal growth factor receptor (EGFR), vascular endothelial growth factor (VEGFR‐2), and Topo II. The triazole ring aligns the pharmacophores, facilitating binding to multiple targets. The compounds exhibited low micromolar cytotoxicity and high selectivity, with **20**, in particular, standing out as a potent VEGFR‐2 (IC_50_ = 0.069 µM), moderate EGFR inhibitor (IC_50_ = 0.103 µM), and potent Topo II inhibitor (IC_50_ = 19.74 µM). Molecular studies have shown that **20** arrests the cell cycle in the G2/M phase and increases apoptosis. Molecular docking studies of the **20** revealed favorable binding interactions within the active sites of EGFR, VEGFR‐2, and Topo II. This study highlights the importance of the triazole ring obtained via CuAAC in multitarget drug design.^[^ ^126^ ^]^


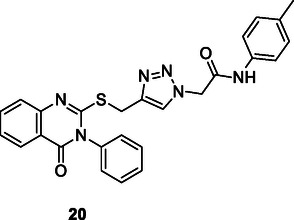


Costa et al. obtained a series of triazole compounds by conducting the CuAAC reaction between 2‐azidobenzaldehyde and alkynes. The binding affinities of these synthesized compounds for AD‐related targets, such as BACE, glycogen synthase kinase‐3β (GSK‐3β), and AChE enzymes, were assessed using molecular docking, and their antioxidant effects were also examined. The compound **21** bearing 4‐CH_3_Ph substituent was found to reverse cognitive decline and memory loss by modulating intracellular mechanisms disrupted in AD, including AChE activity, the amyloid cascade, and GSK‐3β expression in a streptozocin‐induced mouse model.^[^ ^127^ ^]^ This study highlights that compounds capable of interacting with multiple targets can be effectively synthesized through the CuAAC process.


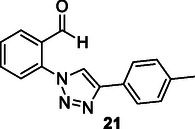


### 9.2 Suzuki Cross‐Coupling Reaction

**Scheme 2:**
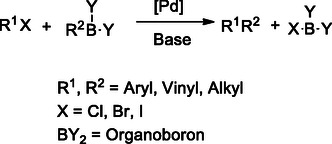
Palladium catalyzed Suzuki cross‐coupling reaction.

The Suzuki cross‐coupling reaction is a widely used method for forming carbon–carbon (C─C) bonds between organic electrophiles (R_1_─X), such as halides or pseudohalides, and organoboron compounds (R_2_‐BY_2_), typically in a basic medium, under palladium catalysis (**Scheme** **2**).^[^ ^128^ ^]^ Generally carried out at 60–80 °C with high efficiency,^[^ ^129^ ^]^ this reaction is particularly valuable for building biaryl frameworks and extended conjugated systems, which are frequently associated with enhanced π–π stacking interactions with biological targets, such as kinases or DNA.^[^ ^130^ ^]^

Hou et al. developed a seven‐step reaction protocol to synthesize compound **22**, a multitarget receptor tyrosine kinase inhibitor, which exhibits significant inhibitory activity, starting from 3‐fluoroaniline. The Suzuki cross‐coupling reaction was employed as a key step in the synthesis of **22**. This reaction offers advantages, including simplicity, high yield, and purity, making it suitable for industrial production.^[^ ^131^ ^]^

**Scheme 4:**
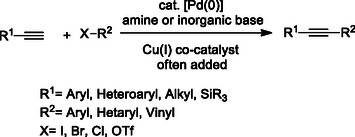
Sonogashira reaction procedure.


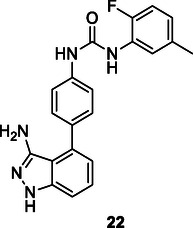


Parveen et al. synthesized a series of *tert*‐butyl 2‐(4‐(benzen‐1‐yl)phenyl)‐1*H*‐indole‐1‐carboxylate derivatives **23** using the Suzuki cross‐coupling reaction. The synthesized compounds were evaluated for their inhibitory effects on AChE and BuChE enzymes, revealing that both **23a** and **23b** exhibited significant inhibitory properties against these enzymes.^[^ ^132^ ^]^


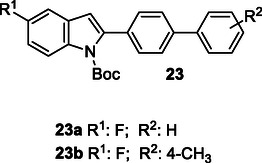


### 9.3 Stille Reaction

**Scheme 3:**
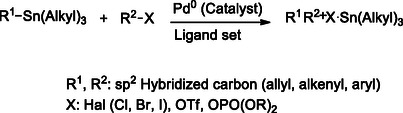
Stille reaction procedure.

The Stille reaction is a palladium‐catalyzed cross‐coupling process that enables the formation of carbon–carbon (C─C) bonds between alkyl or aryl halides and organostannanes, such as alkenyl, aryl, or alkynyl tin compounds (**Scheme** **3**).^[^ ^133^ ^]^ This reaction is particularly advantageous for constructing complex heteroaryl frameworks due to its broad functional group tolerance and compatibility with a wide range of substrates. The mild reaction conditions, along with the oxygen‐ and moisture‐stability of organostannanes, further enhance its utility in the synthesis of structurally intricate molecules.^[^ ^134^ ^]^

Rasolofonjatovo et al. synthesized a series of compounds **24** by combining the cores of combretastatin A‐4 (CA‐4) and isocombrestatin A‐4 (isoCA‐4) via the Stille reaction. The resulting compounds, which are analogs of CA‐4 and isoCA‐4, were tested for their ability to inhibit cell growth and their antitubulin activity. While some compounds exhibited antitubulin activity, they showed lower cytotoxicity than CA‐4 and isoCA‐4. Nevertheless, some of these molecules still demonstrated promising antitubulin activity.^[^ ^135^ ^]^


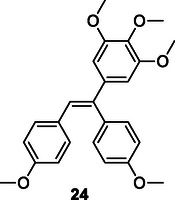


Kumar Dey et al. reported a versatile method using the Stille reaction for the total synthesis of (*S*)‐Zearalenone and (*R*)‐de‐*O*‐methyllasiodiplodin,^[^ ^136^ ^]^ derivatives of two natural compounds, Zearalenone and Lasiodiplodin,^[^ ^137^ ^]^ which exhibit antimicrobial, antileukemic, antibacterial, estrogenic, uterotropic, and prostaglandin inhibitory activities.


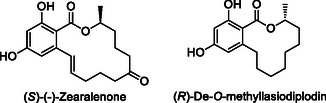


### 9.4 Sonogashira Reaction

The Sonogashira reaction is a cross‐coupling reaction that enables the coupling of terminal acetylenes and aryl or vinyl halides using palladium or other transition metal catalysts (**Scheme** **4**). The Sonogashira reaction is widely used in organic synthesis to form sp^2^–sp carbon–carbon bonds. This reaction is particularly favored in the synthesis of natural products, conjugated polymers, dendrimers, heterocycles, and biologically active compounds.^[^ ^138^ ^]^

Khatyr et al. synthesized multitarget ligands **25** using the chemoselective palladium‐catalyzed Sonogashira reaction. The method offers significant adaptability and synthetic versatility, enabling the production of multitopic metal‐binding compounds with phenyl substituents and various bridging units.^[^ ^139^ ^]^


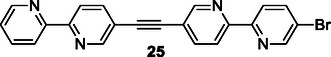


### 9.5 Heck Reaction

**Scheme 5:**
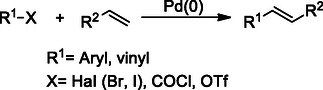
Heck reaction procedure.

The Heck reaction facilitates the formation of substituted alkenes through the interaction of an activated alkene and an unsaturated halide, in the presence of a palladium catalyst and a base (**Scheme** **5**). The chemoselectivity, mild reaction conditions, trans‐selective nature, and low cost and toxicity of the reagents make the Heck reaction attractive for synthetic applications. The reaction is compatible with a wide range of functional groups, including aldehydes, amines, and esters, and yields high outputs.^[^ ^140^ ^]^

Synthetic indole derivatives **26** produced via the Heck reaction exhibit anticancer properties by inhibiting tubulin polymerization, HDAC, DNA topoisomerase, sirtuin, and sigma receptors in cancer cells.^[^ ^89^ ^–^ ^91^ ^]^


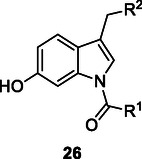


### 9.6 Diels–Alder Reaction

**Scheme 6:**
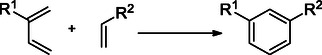
Diels–Alder reaction procedure.


The Diels–Alder reaction (**Scheme** **6**) is a [4+2] cycloaddition that allows the formation of substituted cyclohexene derivatives by reacting a conjugated diene with a substituted alkene (dienophile).^[^ ^141^ ^]^ Arepalli et al. synthesized a series of compounds **27**, including the 1,3‐diphenylbenzo[*f*][1,7]benzonaphthyridine derivative, which exhibits cytotoxic properties and acts as a topoisomerase II‐α inhibitor, through an intermolecular imino Diels–Alder reaction. The Diels–Alder reaction provides advantages, such as regioselectivity, cost‐effectiveness, multicomponent capability, and a broad substrate scope.^[^ ^142^ ^]^

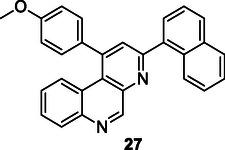


### 9.7 Biginelli Reaction

**Scheme 7:**
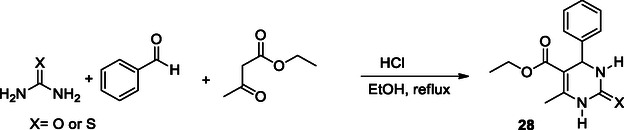
Biginelli reaction procedure for compound **28.**

The Biginelli reaction is a cyclocondensation reaction in which β‐keto ester, an aromatic aldehyde, and thiourea or urea react in one‐pot under acidic conditions to form 3,4‐dihydropyrimidin‐2(1*H*)‐ones/thiones **28** (**Scheme** **7**).^[^ ^143^ ^]^ The formation of 3,4‐dihydropyrimidine‐2(1*H*)‐ones/thiones and their derivatives, which exhibit various pharmacological effects such as antibacterial,^[^ ^144^ ^]^ anticancer,^[^ ^145^ ^]^ antifungal,^[^ ^146^ ^]^ urease enzyme inhibition,^[^ ^147^ ^]^ antioxidant,^[^ ^148^ ^]^ and anti‐HIV^[^ ^149^ ^]^ activities, suggests that the Biginelli reaction is a valuable tool in the synthesis of multitarget drugs.

Malek et al. designed and synthesized a series of compounds **29** using the Biginelli reaction. The synthesized compounds **29a** (BuChE IC_50_: 4.78 ± 0.58 µM, AChE IC_50_: 342  ± 64 nM, calcium antagonism (% Inhibition at 10 µM): 67 ± 10, ORAC: 1.60 ± 0.1, **29b** (BuChE IC_50_: 6.63 ± 0.71 µM, AChE IC_50_: 462 ± 40 Nm, calcium antagonism (% Inhibition at 10 µM): 50 ± 13, ORAC: 1.85 ± 0.16, and **29c** (BuChE IC_50_: 5.43 ± 0.24 µM, AChE IC_50_: 352 ± 15 nM, calcium antagonism (% Inhibition at 10 µM): 74 ± 16, ORAC: 1.41 ± 0.06) were found to exhibit cholinesterase inhibition, calcium channel blockade, antioxidant activity, and the ability to activate Nrf2‐ARE pathways. Moreover, compounds **29a–** **c** were identified as promising multitarget agents for further investigation in the treatment of AD.^[^ ^150^ ^]^


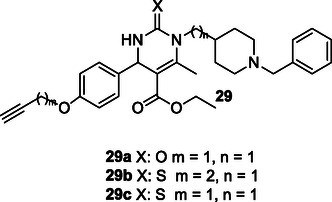


## 10 Where Multitarget Medications are Most Effective?

Neurodegenerative diseases encompass a broad range of neurological disorders, all of which are characterized by the progressive degeneration of neurons in either the central or peripheral nervous system.^[^ ^151^ ^]^ These conditions have a profound impact, significantly impairing the quality of life for millions of individuals worldwide while also imposing a considerable socioeconomic burden on society.^[^ ^152^ ^]^ Numerous studies have been conducted to investigate the pathologies underlying neurodegenerative diseases; however, the primary causes of these disorders remain insufficiently defined.^[^ ^153^ ^]^ Common pathological factors, including oxidative stress, neurotransmitter deficiencies, imbalances in metal ions, and proteopathy, are prevalent across many neurodegenerative diseases, contributing to their pathogenesis.^[^ ^154^ ^]^ It has been proposed that these pathological factors are interrelated and collectively drive disease progression.^[^ ^155^ ^]^

**Figure 12:**
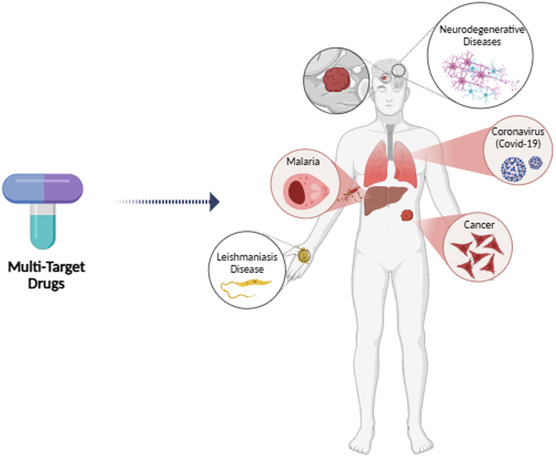
Some uses of multitargeted durgs. https://www.biorender.com/.

Pharmaceuticals approved by the EMA and FDA for treating certain neurodegenerative disorders typically target a singular pathological mechanism, providing symptomatic relief (**Figure** **12**). However, these drugs do not offer a cure for diseases, such as Alzheimer's, Parkinson's, schizophrenia, and ALS, nor can they prevent disease progression. As a result, the development of molecules that can modulate multiple targets, addressing several pathological processes simultaneously, has been proposed as a promising strategy for combating the complex pathologies associated with neurodegenerative diseases.^[^ ^68^ ^]^

Importantly, the clinical adoption of multitarget strategies is gaining momentum. In Germany, ≈20% of newly approved drugs are classified as multitarget therapies, either as fixed‐dose combinations or as agents with well‐defined polypharmacological profiles.^[^ ^8^ ^]^ This trend reflects an increasing recognition of polypharmacology as a viable and effective approach for treating complex neurological conditions.

### 10.1 AD

AD, first identified by Alois Alzheimer in 1906, is a progressive neurodegenerative condition characterized by a decline in cognitive function, memory, and the ability to perform daily activities.^[^ ^156^ ^]^ Histopathological examination reveals the deposition of Aβ peptides, which form amyloid plaques in the extracellular space.^[^ ^157^ ^]^ Additionally, the disease is marked by the accumulation of intracellular neurofibrillary tangles, resulting from the hyperphosphorylation of the microtubule‐associated protein tau, along with notable neuronal and synaptic degeneration.^[^ ^158^ ^]^ AD leads to a range of neuropsychiatric symptoms and behavioral disorders, contributing to cognitive decline in affected individuals.^[^ ^159^ ^]^

Numerous studies have explored the mechanisms and etiology of AD, uncovering multiple factors that contribute to its development.^[^ ^25^ ^]^ These factors include reduced acetylcholine concentrations, accumulation of Aβ peptides, hyperphosphorylation of tau protein, dysregulation of redox metal ions, oxidative stress, and neuroinflammation.^[^ ^160^ ^]^ Currently, drugs such as rivastigmine and memantine are used to treat AD, but they only target individual mechanisms associated with the disease, resulting in limited symptomatic relief.^[^ ^161^ ^]^ Therefore, the design of molecules that address multiple mechanisms involved in AD may offer a promising strategy for more effective treatment options.^[^ ^162^ ^]^

A series of compounds were synthesized by Bolea et al. by linking the benzylpiperidine moiety of the AChE inhibitor donepezil with the indolyl propargylamino moiety of the MAO inhibitor *N*‐[(5‐benzyloxy‐1‐methyl‐1*H*‐indol‐2‐yl)methyl]‐*N*‐methylprop‐2‐yn‐1‐amine via an oligomethylene linker. The synthesized compound exhibited potent inhibitory activity against both MAO‐A (IC_50_: 5.2 ± 1.1 nM) and MAO‐B (IC_50_: 43 ± 8.0 nM), along with modest inhibitory activity toward AChE (IC_50_ = 0.35 ± 0.01 μM) and BuChE (IC_50_ = 0.35 ± 0.01 μM). As a result, they hypothesized that compound **30** could be a viable multitarget therapeutic candidate for AD.^[^ ^163^ ^]^


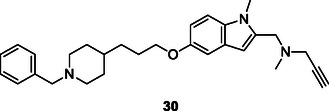


Simoni et al. synthesized a series of multitarget molecules incorporating galantamine and memantine, both FDA‐approved drugs used in AD treatment. The compounds were evaluated for their AChE inhibitory activity and binding affinity to the *N*‐methyl‐*D*‐aspartate receptor (NMDAR), demonstrating both AChE inhibitory activity and affinity for NMDAR. Furthermore, the compounds showed selectivity toward the 2B subunit of NMDAR (NR2B), with certain compounds exhibiting notable affinity for NR2B. Compound **31**, named memagal, was found to block NMDA‐induced neurotoxicity (IC_50_: 0.28 nM) and exhibited neuroprotective properties.^[^ ^164^ ^]^


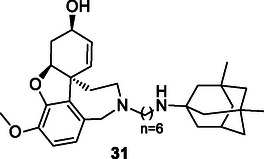


Huang et al. designed and synthesized a series of compounds derived from benzylidenin to obtain multitarget agents for AD. Most of these compounds demonstrated MAO‐B inhibition (IC_50_: 7.50 µM) and self‐induced Aβ_1‐42_ aggregation (80.1% at 20 µM). Additionally, the compounds exhibited metal chelation and antioxidant properties (ORAC‐FL value of 5.60, equivalent to trolox). Notably, compound **32** showed the highest activity and could be explored as a multitarget molecule for AD treatment.^[^ ^165^ ^]^


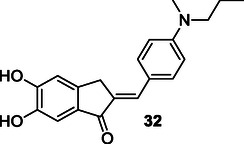


Lu et al. synthesized various resveratrol‐based compounds **33** aimed at treating AD. The synthesized compounds were found to inhibit both self‐induced and Cu(II)‐induced Aβ_1‐42_ aggregation. Additionally, these compounds demonstrated potential antioxidant and biometal chelator activity. Compounds **33a** with dimethoxy substituent (Aβ aggregation IC_50_: 7.56 μM, MAO‐A IC_50_: 8.19 μM, MAO‐B IC_50_: 12.16 μM, AChE IC_50_: 36.04 μM) and **33b** with two hydroxyl groups (Aβ aggregation IC_50_: 6.51 μM, MAO‐A IC_50_: 7.08 μM, MAO‐B IC_50_: 14.09 μM, AChE IC_50_: 6.27 μM) were identified as potential lead compounds for AD treatment.^[^ ^166^ ^]^


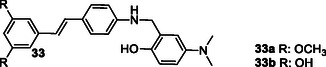


Luo et al. synthesized compounds that combine donepezil, a cholinesterase inhibitor, with ebselen, an antioxidant. Compound **34** exhibited glutathione peroxidase‐like activity (123.5 μM min^−1^), and inhibited both AChE (IC_50_: 42 nM for eeAChE and 97 nM for human acetylcholinesterase [HuAChE]) and BuChE, as well as Aβ aggregation induced by AChE.^[^ ^167^ ^]^


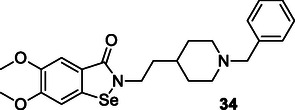


Thiratmatrakul et al. synthesized three new tacrine‐carbazole hybrids **35** that exhibited strong AChE inhibitory activity and higher selectivity for AChE over BuChE. Compound **35a** (AChE IC_50_: 0.48 ± 0.14 µM, BuChE IC_50_: 52.14 ± 6.08 µM), which has a methoxy group in its structure and a shorter alkyl chain, showed higher AChE activity compared to **35b** (AChE IC_50_: 0.95 ± 0.27 µM, BuChE IC_50_: 19.37 ± 0.54 µM) and **35c** (AChE IC_50_: 1.03 ± 0.23 µM, BuChE IC_50_: 64.32 ± 11.92 µM), while compound **35b**, which has a methoxy group in its structure and a longer alkyl chain, showed the highest BuChE enzyme activity. These compounds also exhibited radical scavenging and neuroprotective activities against both H_2_O_2_‐induced oxidative stress and Aβ_1‐42_ toxicity.^[^ ^168^ ^]^


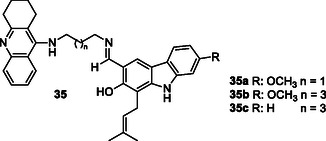


Sang et al. designed a series of felluric acid derivatives using a multitarget ligand approach to treat AD. In vitro investigations revealed that all synthesized compounds were extremely potent and selective inhibitors of BuChE. Compound **36** had the most effective BuChE (IC_50_ = 0.0089 μM) inhibition and also demonstrated inhibitory capabilities for MAO‐A (IC_50_ = 6.3 μM) and MAO‐B (IC_50_ = 8.6 μM). Compound **36** was found to inhibit and degrade self‐induced Aβ aggregation (53.9%), demonstrate antioxidant properties, provide neuroprotection against Aβ_1‐42_‐induced (43.8%) SH‐SY5Y neurotoxicity, and function as an autophagic activator. This investigation indicates that compound **33** could be a multifunctional candidate against AD.^[^ ^169^ ^]^


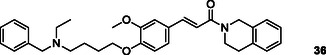


Kaur et al. designed and synthesized triazole derivatives that target multiple mechanisms involved in AD. Compound **37** exhibited strong inhibition of self‐induced Aβ42 aggregation (78.02%, IC_50_: 4.578 ± 0.109 μM) and disrupted preformed Aβ42 aggregates. In addition, compound **37** demonstrated metal‐chelating properties, preventing ROS formation in the copper‐ascorbate redox cycle, indicating its potential as a multitarget compound for AD.^[^ ^98^ ^]^


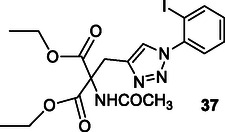


Frang et al. modified the ketone carbonyl group of donepezil to create hybrid compounds combined with nonsteroidal anti‐inflammatory drugs. Compound **38** exhibited a tenfold greater inhibition of AChE (IC_50_: 0.015 µM) and BuChE (IC_50_: 0.80 µM) than donepezil. These compounds also inhibited COX‐1 and COX‐2, while preventing the secretion of proinflammatory cytokines (TNF‐α and Interlökin‐1β). Furthermore, compound **38** exhibited neuroprotective properties against Aβ‐induced toxicity.^[^ ^170^ ^]^


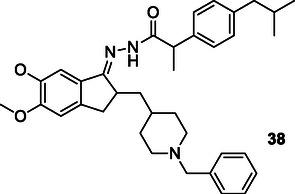


Zhang et al. designed and synthesized, eighteen new bakuchiol derivatives as multitargeted anti‐Alzheimer's drugs. Most of the compounds exhibited AChE and BuChE activity in varying degrees, among them, compound **39** and **40** showed relatively strong potent AChE inhibitory activities IC_50_ = 32.07 ± 2.00 μM and IC_50_ = 34.78 ± 0.34 μM, respectively. Better activities were reported for the compound **39** with substitution at 7‐position of the coumarin and compound **40** with substitution at 7‐position of the coumarin; also, the AChE‐inhibition activities of those compounds were influenced by the length of the linker between the coumarin and bakuchiol.^[^ ^171^ ^]^


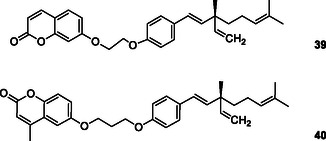


Ceyhan et al. prepared indolyl imine derivatives, as multitarget agents via Schiff base reaction, and investigated their in vitro antidiabetic activities, anticholinesterase potency, and antioxidant properties. The anticholinesterase potency of the compounds was investigated toward the AChE and BuChE enzymes. The findings indicate that the compounds demonstrate a moderate level of efficacy against the BuChE enzyme, with the most effective inhibition concentration of 30.48 μM for compound **41**.^[^ ^172^ ^]^


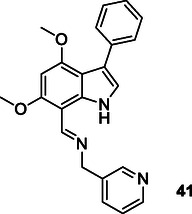


Dixit and Prabhu used pyridine and chalcone to obtain multifunctional molecules for AD. The designed compounds showed remarkable potential for AChE activities. All the compounds showed mixed inhibition of AChE in enzyme kinetic studies. They found that IC_50_ values to the standard Tacrine ranging from 0.1 to 0.44 μM where **42** with the benzyloxy substitution had the best IC_50_ values with 0.1 ± 0.01 μM.^[^ ^173^ ^]^


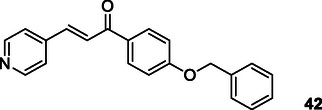


Luo et al. designed, synthesized and evaluated Scutellarein hybrids as multifunctional therapeutic agents for the treatment of AD. Reports indicate that certain target compounds demonstrate moderate to potent inhibition of human AChE (HuAChE), with IC_50_ values ranging from 8.91 to 16.52 μM, whereas their inhibitory potency against *hu*BuChE appears to be comparatively weak. Compound **43** with a N(C_2_H_5_)_2_ and a phenyl group exhibited the most potent inhibition of *hu*AChE enzymes with IC_50_ values 8.91 ± 0.08 μM.^[^ ^174^ ^]^


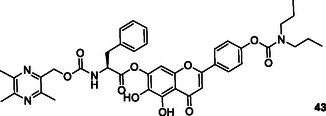


Long et al. designed and synthesized series of novel hybrid compounds connecting capsaicin and tacrine moieties as multitarget drugs to treat AD. The biological assays indicated that most of these compounds demonstrated strong inhibition of AChE and BuChE activities with IC_50_ values, as well as good blood–brain barrier permeability. The researchers determined most of the synthesized compounds exhibit excellent inhibitory activity against AChE and BuChE. According to results, compound **44** exhibits the strongest AChE inhibitory activity with IC_50_ = 69.8 nM whilst compound **45** exhibits the strongest BuChE inhibitory activity with IC_50_ = 45.0 nM.^[^ ^175^ ^]^


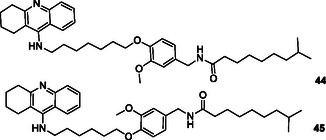


Tamaddon‐Abibigloo et al. designed and synthesized s‐triazine, isatin, and aniline hybrids **46** as MTDL and investigated in vitro and in silico anti‐AD activity. Biological evaluation showed that these molecules were excellent inhibitors with IC_50_ values ranging from 0.2 to 734.5 nM for AChE, and 0.02 to 1.92 μM for BuChE. Compounds, **46a** with IC_50_ AChE = 0.7 nM and IC_50_ BuChE = 0.09 μM, and **46b** with IC_50_ AChE = 0.2 nM and IC_50_ BuChE = 0.03 μM were the most potent compounds. They also reported that *N*‐benzyl substituent bonded to isatin and 2‐OMe or 2‐OH groups attached to the aniline moiety possessed good antioxidant activity with EC_50_ values ranging from 64.2 to 103.6 μM.^[^ ^176^ ^]^


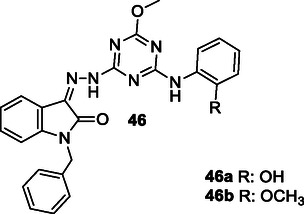


Madhav et al. described pyrazine‐based MTDL **47** for the dual inhibition of *tau*‐aggregation and *HuAChE.* Compounds **47a** and **47b** exhibit remarkable inhibition of AChE with an IC_50_ value of 0.71 and 1.09 μM, respectively. Also, these two compounds were also remarkably able to inhibit *tau*‐oligomerization with an EC_50_ value of 2.21 and 2.71 μM, respectively, and were marked as the most potent MTDLs in this research.^[^ ^177^ ^]^


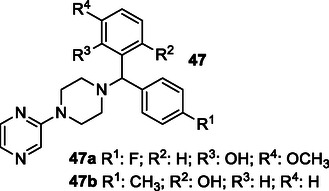


Waly et al. synthesized a series of integrated anti‐AD multitargeted ligands **48** bearing a pyrazolopyridine scaffold. Among them, compound **48a** bearing a bromo substituent had a significant effect on *Hu*AChE (IC_50_ = 0.17 μM) and *h*BuChE (IC_50_ = 0.16 μM), and compound **48b**, bearing a dimethoxy arrangement, exhibited strong inhibitory activity against *Hu*AChE (IC_50_ = 0.17 μM) and *h*BuChE (IC_50_ = 0.69 μM). When compared with the reference rivastigmine (IC_50_ = 1.32 μM), compound **48a** showed eightfold higher activity and compound **48b** showed twofold higher activity against *h*BuChE.^[^ ^178^ ^]^


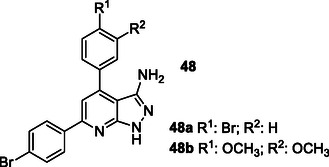


### 10.2 Parkinson's Disease

Parkinson's disease, the second most prevalent neurodegenerative disorder following AD, is a progressive condition marked by the degeneration of dopaminergic neurons in the substantia pars compacta and the presence of intraneuronal clumps known as Lewy bodies. The degeneration of dopaminergic neurons leads to symptoms including uncontrollable resting tremors, bradykinesia, and postural instability in patients. Furthermore, various neurotransmitter systems contribute to Parkinson's disease, resulting in the degeneration of cholinergic, serotonergic, and noradrenergic neurons, which manifests as cognitive impairment, sleep disturbances, and depression.^[^ ^179^ ^]^

Parkinson's disease, characterized by its intricate pathology, arises from a multitude of molecular processes and pathways, including the ubiquitin‐proteasomal system, the autophagy‐lysosomal pathway, the aberrant aggregation of α‐synuclein, neuroinflammation, mitochondrial dysfunction, and oxidative stress.^[^ ^180^ ^]^ Levodopa (L‐Dopa), a dopamine precursor; pramipexole and ropirinol, dopamine agonists; selegiline, an MAO inhibitor; and entacapone and tolcapone, which are catechol ortho methyltransferase inhibitors, are used in the treatment of Parkinson's disease.^[^ ^181^ ^]^ Given that these compounds interact solely with a singular pathogenic pathway, they are incapable of impeding or halting the advancement of the disease.^[^ ^182^ ^]^ The intricate origins of Parkinson's disease, coupled with the reality that existing medications merely mitigate symptoms, underscore the necessity for the development and synthesis of compounds that engage with multiple targets in the pursuit of effective treatment for the condition.^[^ ^183^ ^]^

Sashidhara et al. synthesized a series of 3‐arylcoumarin‐tetracyclic tacrine derivatives **49** capable of interacting with multiple targets for the treatment of Parkinson's disease. Compounds **49a** and **49b** exhibited significant antioxidant activities and reduced the aggregation of alpha‐synuclein protein.^[^ ^184^ ^]^


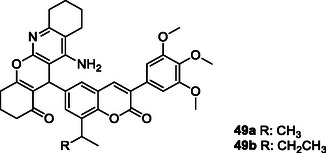


Hagenow et al. designed the compounds that can interact with A1R/A2AR and histamine H3 receptor (H3R) by overlapping the piperidino‐/pyrrolidino phenyl H3R pharmacophore with the adenosine antagonist arylindenopyrimidine pharmacophore group, taking advantage of the ‘caffeine‐like effects’ of adenosine A1/A2A receptor (A1R/A2AR) antagonists along with the arousal‐promoting properties of histamine H3R antagonists. Compounds **50** and **51** mitigated L‐Dopa‐induced dyskinesia, while compound **51** also prolonged wakefulness.^[^ ^185^ ^]^


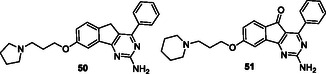


Song et al. designed and synthesized a series of 6‐benzyloxyphthalide derivatives **52** with antioxidant, antineuroinflammatory, and MAO‐B inhibitory characteristics. Compounds **52a** (IC_50_ = 1.33 nM, SI = 865) and **52b** (IC_50_ = 0.02 nM, SI = 40.250) showed strong MAO‐B inhibition and modest antioxidant (0.34 and 0.36 Trolox equivalent, respectively) activity. Furthermore, in additional research, compounds **52a** and **52b** were identified as competitive and semi‐reversible MAO‐B inhibitors.^[^ ^186^ ^]^


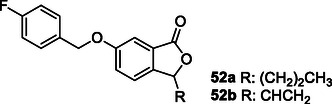


Cao et al. designed and synthesized a series of 2‐hydroxyl‐4‐benzyloxybenzyl aniline derivatives that can interact with multiple targets to treat Parkinson's disease. The synthesized compound **53** was found to have MAO‐B (IC_50_: 0.014 µM) inhibition, metal chelation, neuroprotective effect, and high‐level antioxidant activity properties. Compound **53** was also found to reduce neuroinflammation by inhibiting the nuclear factor kappa B pathway. In addition, compound **53** had the ability to improve Parkinson's disease symptoms caused by 1‐methyl‐4‐phenyl‐1,2,3,6‐tetrahydropyridine in mice by improving dopamine levels and suppressing oxidative damage (ORAC: 2.14 Trolox equivalent).^[^ ^187^ ^]^


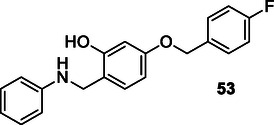


### 10.3 ALS

ALS is a neurodegenerative disorder marked by the degeneration of lower and upper motor neurons, resulting in muscle atrophy, paralysis, and respiratory failure within 3–5 years postdiagnosis, ultimately culminating in the patient's death.^[^ ^188^ ^]^ Edaravone, an excitotoxic agent, and Riluzole, a radical scavenger, are utilized in the management of ALS, offering very modest clinical relief to patients. The primary reason for this is because ALS possesses a multifaceted etiology.^[^ ^189^ ^]^ The advancement and progression of ALS result from multiple pathogenic pathways, including oxidative stress, mitochondrial malfunction, glutamate‐induced excitotoxicity, apoptosis, neuroinflammation, and axonal degradation.^[^ ^190^ ^]^ Furthermore, cell groups outside motor neurons, including microglia, astrocytes, macrophages, and oligodendrocytes, contribute to the pathogenesis of ALS, creating a more intricate landscape.^[^ ^191^ ^]^ The multifactorial and complex molecular causes of ALS suggest that polypharmacology may be a promising treatment approach.^[^ ^192^ ^]^

Albertini et al. designed and synthesized a series of compounds that can interact with multiple targets by combining the pharmacophore groups of riluzole and rasagiline compounds for the treatment of ALS. Synthesized compound **54** was found to have MAO‐A (IC_50_ = 6.9 μM) inhibition, neuroprotective, and neuroinflammatory properties.^[^ ^192^ ^]^


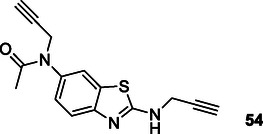


Martín‐Cámara et al. designed and synthesized hybrid compounds containing the pharmacophores of Rho kinase inhibitor fasudil and NRF2 inducers felluric and caffeic acid by utilizing molecular docking and molecular mechanics studies. Compound **55** was selected due to its high multitarget effect and good tolerability. The selected compound was found to increase the expression of antioxidant response (EC_50_: 9.68 µM) enzymes Heme oxygenase‐1 and NAD(P)H dehydrogenase quinone 1 through a mechanism dependent on cap’n’collar hemology (ECH)‐associated protein 1 and to induce NRF2 (CD: 1 µM) signaling. Analysis of mRNA and protein levels of the NRF2 pathway revealed that compound **55** controlled NRF2 signaling and induced superoxide dismutase 1 in ALS lymphoblasts, but did not induce it in sALS, which was increased in the basal state. Compound **55** has a therapeutic effect in ALS patients with SOD1 mutation.^[^ ^193^ ^]^


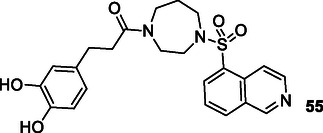


### 10.4 Cancer

Cancer is a complex disease driven by multiple factors, including genetic and cellular mutations that result in the unregulated proliferation of cells.^[^ ^194^ ^]^ While the evolution and advancement of cancer encompass various receptors and signaling pathways, most pharmacological interventions currently used in treatment are designed to target a single molecular entity.^[^ ^195^ ^]^ A major challenge in modern cancer therapy is the emergence of treatment resistance.^[^ ^196^ ^]^ This resistance arises due to several factors, including the overexpression of antiapoptotic proteins, mutations in key signaling molecules, and increased expression of drug efflux pumps.^[^ ^197^ ^]^ These complexities not only hinder effective cancer treatment but also contribute to disease recurrence.^[^ ^198^ ^]^ The intricate cellular mechanisms of cancer, the development of resistance to pharmacological agents, and the regeneration of neoplastic tissues present significant challenges to successful therapy. In response, researchers are increasingly exploring pharmacological agents that interact with multiple targets rather than single‐target approaches. This shift aims to overcome the limitations of conventional chemotherapy and facilitate the development of safer and more effective therapeutic strategies.^[^ ^199^ ^]^

Ning et al. designed and synthesized a series of macrocyclic compounds that can simultaneously inhibit HDAC, FMS‐like tyrosine kinase 3 (FLT3) and JAK2. Most of the synthesized macrocyclic compounds showed strong HDAC (IC_50_: 87 nM), FLT3 (IC_50_: 87 nM) and JAK2 (IC_50_: 686 nM) inhibition properties under both cell‐free and cellular conditions. In vitro assays showed that the compounds were more cytotoxic to MV4‐11 and HEL cells carrying FLT3 internal tandem duplication (FLT3‐ITD) and JAK2^V617F^ mutation. Compound **56** could be an anticancer drug with possible treatment for FLTD3‐ITD or JAK2V617F positive acute myeloid leukemia patients.^[^ ^200^ ^]^


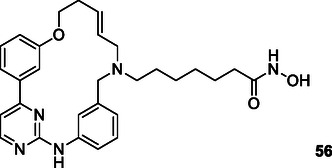


Zang et al. designed and synthesized pazopanib hybrid series as polypharmacological antitumor agents based on the crosstalk between HDAC and VEGFR pathways. The synthesized compounds **57** (HDAC IC_50_: 11.3 µM VEGFR‐2 inhibition rate at 0.2 µM: 100%) and **58** (HDAC IC_50_: 0.0033 µM VEGFR‐2 inhibition rate at 0.2 µM: 93%) demonstrated HDAC and VEGFR‐2 inhibition properties, along with significant antiproliferative and tyrosine kinase inhibitory activity.^[^ ^201^ ^]^


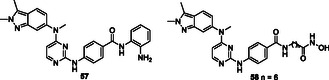


Liu et al. designed a series of styrylbenzylsulfone derivatives, resulting from the platinum‐based modification of the Rigosertib compound's side chain. Compound **59** exhibited anticancer activity that was 1000 times greater than that of cisplatin against the evaluated cell lines and multidrug‐resistant cells. Compound **59** directly interacts with CRAF and inhibits its activation, hence disrupting the signaling pathway between RAS and CRAF. The elevation of platinum levels within the cell renders compound **59** more efficacious than cisplatin inducing DNA damage, promoting the buildup of ROS, and diminishing mitochondrial membrane potential. Furthermore, it was proven that compound **59** promoted endogenous apoptosis and suppressed tumor growth in a preexisting A549 cancer xenograft model without exhibiting adverse effects.^[^ ^202^ ^]^


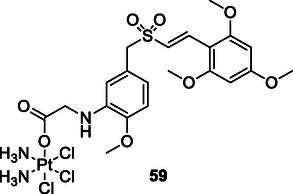


Peng et al. designed and synthesized a series of compounds capable of inhibiting HDAC3 and tubulin by integrating the pharmacophore groups of 4‐substituted methoxybenzoyl‐aryl‐thiazoles (SMART), a tubulin inhibitor, with MS‐275, an HDAC inhibitor. Compound **60** demonstrated high HDAC3 (IC_50_: 30 nM) and tubulin inhibitory (IC_50_: 12.2 µM) characteristics, while also demonstrating high antiproliferative activities against several cancer cell lines. Furthermore, the compound **60** exhibited superior tumor suppression characteristics compared to the combination of SMART and MS‐275 in the melanoma tumor model. Compound **60** was shown to exhibit safe cardiotoxicity without inducing nephrotoxicity or hepatotoxicity. The results suggest that compound **60** may be the lead compound as an anticancer agent that can interact with multiple targets.^[^ ^203^ ^]^


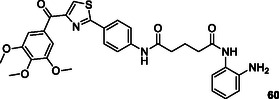


Tilekar et al. designed and synthesized a series of compounds **61** that can simultaneously interact with peroxisome proliferator‐activated receptor gamma (PPARγ) and HDAC. The compound **61a** showed the highest activity against PPARγ (EC_50_: 0.245 μM) and HDAC4 (IC_50_:1.1 ± 1.7 μM), while compounds **61a** and **61b** showed cytotoxic activity against CCRF‐CEM cells, apoptosis‐inducing effect, and caused DNA fragmentation. In addition, compound **61b** was found to modulate c‐Myc gene expression, cleave caspase‐3, and cause in vivo tumor regression in CCRF‐CEM tumor xenografts.^[^ ^204^ ^]^


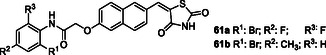


Wang et al. designed and synthesized compound **62**, which is an inhibitor of PARP1/2 (IC_50_: 13 nM) and bromodomain‐containing protein 4 (BRD4) (IC_50_: 75 nM). The synthesized compound was found to stop cell cycle progression, inhibit DNA damage repair, and promote autophagy‐associated cell death in pancreatic cancer cells and xenografts. Furthermore, compound **62** was observed to reverse the acceleration of cell cycle progression and the recovery of DNA repair induced by olaparib.^[^ ^205^ ^]^


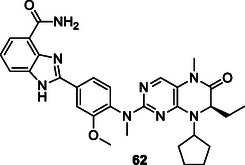


Theodore et al. designed and synthesized a series of new quinoxalinone‐based pyrazole derivatives **63** based on a multitarget‐directed drug design technique to inhibit the antiproliferation of the human cancer cell lines MCF‐7 (breast), HCT‐116 (colon), and A549 (lung). Compounds **63a** and **63b** showed strong anticancer activity. Compound **63a** showed IC_50_ activity of 3.91, 4.13, and 2.86 μM against MCF‐7, HCT‐116, and A549 cancer cell lines, respectively, while compound **63b** showed IC_50_ activity of 2.04, 2.69, and 1.93 μM, respectively. The presence of an electron‐withdrawing nitro group in the pyrazol ring in compound **63b** provided greater potency than the corresponding molecules bearing fluoro, chloro, or bromo groups at the same position of the five membered heterocycle.^[^ ^206^ ^]^


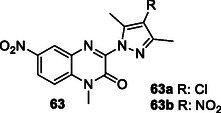


Zhang et al. designed 76 HDAC and receptor tyrosine kinase as multitarget angiogenesis inhibitors **64**. These crucial components involved in angiogenesis, in situ assembly, skeletal transition, molecular hybridization, and pharmacophore fusion. The screening results evaluation of the target compounds indicated that most of the compounds exhibited potent proliferation inhibitory activity on MCF‐7 cell and HT‐29 cells. Results revealed that the series having the highest inhibitory activity 0.072 ± 0.002–6.276 ± 0.832 on MCF‐7 cells. Also, the IC_50_ values of **64a** and **64b** against HT‐29 cells were 0.078 and 0.068 μmol L^−1^, respectively.^[^ ^207^ ^]^


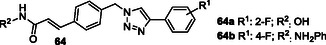


El Hamaky et al. designed and synthesized quinazoline‐1,2,3‐triazole hybrids **65** as multitarget EGFR, VEGFR‐2, and Topo II inhibitors tested them anticancer activity against HeLa, HePG‐2, MCF‐7, and HCT‐116 cancer cell lines. Among them, compounds **65a–c** were the most potent cytotoxic hybrids against all the cancer cells. Compound **65a** with –NO_2_ substituent showed activity against MCF7 (IC_50_: 3.37 μM), HePG2 (IC_50_: 8.18 μM), HeLa (IC_50_: 13.07 μM), and HCT116 (IC_50_: 26.53 μM) while compound **65b** with –CH_3_ substituent displayed activity against MCF7 (IC_50_: 2.57 μM), HePG2 (IC_50_: 5.96 μM), HeLa (IC_50_: 6.41 μM), and HCT116 (IC_50_: 10.63 μM) cells. Also, compound **65c** with 4‐Cl and 4‐Br substituents revealed reasonable activity against MCF7 (IC_50_: 7.33 μM), HePG2 (IC_50_: 8.63 μM), HeLa (IC_50_: 12.91 μM), and HCT116 (IC_50_: 16.12 μM) cells.^[^ ^126^ ^]^


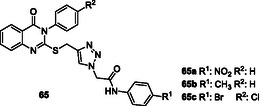


Allawi et al. synthesized indole‐6‐carboxylic acid derivatives to target EGFR and VEGFR‐2 in cancers. Among the compounds, compound **66** with 4‐NO_2_ substituents for targeting EGFR and compound **67** with 2‐Br and 4‐OCH_3_ substituents for targeting VEGFR‐2 exhibited the highest antiproliferation activity. Furthermore, compounds **66** and **67** had the highest EGFR/VEGFR‐2 enzyme inhibitory activity, respectively.^[^ ^208^ ^]^


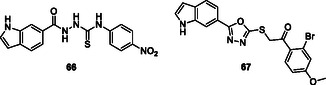


### 10.5 Malaria

Malaria is an illness caused by Plasmodium parasites, which are transmitted to humans through the bite of infected female Anopheles mosquitoes. Four species of Plasmodium within the Plasmodiidae family—*Plasmodium malariae*, *Plasmodium falciparum*, *Plasmodium ovale*, and *Plasmodium vivax*—are known to infect humans and cause malaria. The infection begins in hepatocytes, where parasites undergo replication before being released into the bloodstream, subsequently invading erythrocytes. This results in symptoms including intermittent fever and chills, hemolysis, and, in severe cases, renal failure.^[^ ^209^ ^]^

Many currently available or investigational antimalarial drugs target a single mechanism within the Plasmodium infection cycle.^[^ ^210^ ^]^ However, the therapeutic efficacy of these drugs is often limited due to toxicity, low potency, and the emergence of drug resistance.^[^ ^211^ ^]^ This has facilitated the rapid spread of drug‐resistant Plasmodium strains, particularly in regions where chemotherapeutic interventions are widely implemented.^[^ ^212^ ^]^ The increasing resistance to most antimalarial drugs and the rising incidence of malaria‐related fatalities highlights the urgent need for innovative drug development strategies. Targeting multiple biological pathways represents a promising and effective approach to overcoming these challenges.^[^ ^213^ ^]^

Cysteine protease falcipain‐2 (FP‐2) and dihydrofolate reductase (DHFR) are two enzymes that play important roles in the life cycle of the Plasmodium parasite that causes malaria. Huang et al. designed and synthesized a series of compounds with FP‐2 and DHFR inhibitory properties based on the lead compound **68** (FP‐2 IC_50_: 54.2 µM DHFR IC_50_: 35.5 µM), which was randomly identified as a result of screening for FP‐2 inhibitors. Furthermore, it was found that the FP‐2 and DHFR inhibitory efficacy of the synthesized compound **69** (FP‐2 IC_50_: 7.0 µM DHFR IC_50_: 6.3 µM) was 6–8 times greater than that of compound **68**. This indicates that the synthesized compounds could be formulated as multitarget antimalarial agents.^[^ ^214^ ^]^


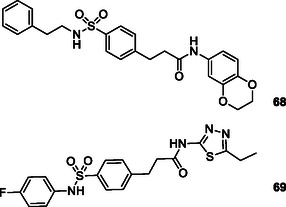


Rawat et al. investigated the inhibitory properties of compounds **70** and **71**, which are recently developed novel permeability pathway inhibitors, against *P. falciparum* dihydroorotate dehydrogenase (PfDHODH) enzyme and cytochrome bc1 complex by using molecular docking and molecular dynamics studies. The analysis revealed that whilst compound **70** inhibited both enzymes, compound **71** reduced PfDHODH via cytochrome bc1. This investigation confirmed that compound **70** could serve as a lead compound in the development of effective multitarget inhibitors for malaria.^[^ ^215^ ^]^


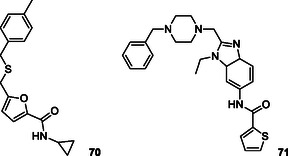


Yang et al. conducted a structure‐based drug design study focusing on the mitochondrial respiratory chain of *P. falciparum*, leading to the identification of molecule **72**. This molecule exhibits the capability to bind concurrently to the allosteric site of type II NADH dehydrogenase and the Qo and Qi sites of cytochrome bc1 in *P. falciparum*. Compound **72** (EC_50_: 0.056 nmol L^−1^) demonstrated efficacy against drug‐resistant strains in vitro and displayed significant solubility alongside in vivo activity.^[^ ^216^ ^]^


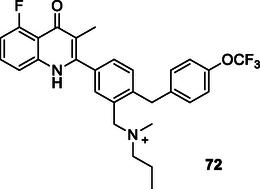


### 10.6 Leishmaniasis Disease

Leishmaniasis is a disease caused by *Leishmania* species, a unicellular parasite transmitted to humans through the bite of sandflies.^[^ ^217^ ^]^ Pentavalent antimony derivatives are employed in the management of leishmaniasis; however, these pharmacological agents exhibit significant toxicity and contribute to the development of resistance in the parasite.^[^ ^218^ ^]^ Amphotericin B and pentamidine‐like drugs are also employed in therapeutic regimens, yet the mechanisms of action of certain commonly used pharmaceuticals remain incompletely understood. Therefore, there is a need to develop compounds that can interact with multiple targets within the parasite while exhibiting minimal toxicity to the patient.^[^ ^219^ ^]^

Mitochondrial iron superoxide dismutase A (FeSODA) and trypanothione reductase (TR) are two enzymes that play crucial roles in the antioxidant defense system of Leishmania donovani. Bora et al. identified compounds **73** (IC_50_: 24.82 ± 0.61 µM) and **74** (IC_50_: 7.52 ± 0.17 µM) that inhibit both FeSODA and TR enzymes through virtual screening. Molecular docking and dynamics studies of the identified compounds revealed that both compounds exhibited activity against FeSODA and TR enzymes. Furthermore, in vitro studies indicated that the compounds inhibited the promastigotes of Leishmania donovani.^[^ ^220^ ^]^


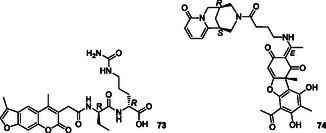


### 10.7 Coronavirus Disease 19 (Covid‐19)

COVID‐19 is a highly contagious and pathogenic viral infection caused by severe acute respiratory syndrome Coronavirus 2 (SARS‐CoV‐2). The disease has led to a considerable number of fatalities worldwide and has escalated into a global pandemic.^[^ ^221^ ^]^ Although COVID‐19 vaccines are widely administered, no fully approved and effective treatments are currently available for the disease.^[^ ^222^ ^]^

RNA viruses, including SARS‐CoV‐2, possess a high genetic variability, enabling them to accumulate genomic mutations. Most contemporary antivirals are designed as single‐target agents, specifically engineered to inhibit enzymes involved in viral interaction, invasion, or replication. However, mutations in these drug targets can reduce the efficacy of existing antiviral therapies. Consequently, there is an urgent need for the development and implementation of drug‐like compounds or approved pharmaceuticals that can modulate multiple targets within SARS‐CoV‐2.^[^ ^223^ ^]^

Chauhan et al. synthesized a series of indolealkylamine derivatives **75** that showed action for three possible COVID‐19‐related proteins, namely melatonin receptors, calmodulin, and human angiotensin converting enzyme 2 (hACE2). Compounds **75a** and **75b** were reported to have strong nanomolar affinity for melatonin receptors, block calmodulin‐dependent calmodulin kinase II activity, and inhibit the binding of SARS‐CoV‐2 spike protein with hCAE2 at micromolar concentration. Both compounds were identified as inhibitors of SARS‐CoV‐2 entry into host cells, with compound **75b** demonstrating a reduction in SARS‐CoV‐2 replication and major protease enzyme activity.^[^ ^224^ ^]^


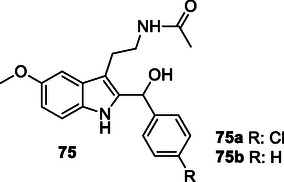


Jeon et al. investigated 48 FDA‐approved drugs against SARS‐CoV‐2. 24 Promising antiviral drug candidates were found against SARS‐CoV‐2 infection; however, certain candidates exhibited minimal inhibitory concentrations, with the FDA‐approved medicines niclosamide and ciclesonide standing out in certain aspects. Niclosamide, an anthelminthic drug, exhibited very potent antiviral activity against SARSCoV‐2 with IC_50_: 0.28 µM. Ciclesonide with IC_50_, 4.33 µM antiviral potency was much lower than niclosamide.^[^ ^225^ ^]^


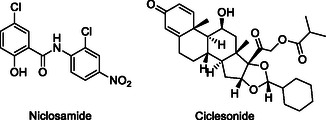


Ren et al. designed multitargeted antiviral drugs with the aim of discovering highly potent SARS‐CoV‐2 inhibitors. The design strategy consists of simultaneously acting on the host ribosome, viral RNA as well as RNA‐dependent RNA polymerases, and nucleocapsid protein of the virus, to impair viral translation, frameshifting, replication, and assembly. For this purpose, three alkaloids, lycorine, emetine, and cephaeline, were selected to potent inhibitors of SARS‐CoV‐2. Lycorine, emetine, and cephaeline alkaloids may inhibit viral protein synthesis. Lycorine, emetine, and cephaeline are alkaloid analogs with structural similarities; however, cephaeline may be better tolerated by patients than emetine, demonstrating comparable efficacy against ZIKV and EBOV infections through interaction with the same drug targets, including the ribosome.^[^ ^226^ ^]^


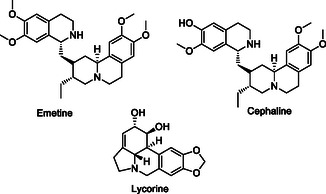


Mun et al. reported the multitarget interaction of coumarins against COVID‐19 using molecular docking analysis. The researchers conducted a multitargeted molecular docking study involving the coumarin drugs phenprocoumon, hymecromone, and psoralen. Researchers revealed that the modified coumarin derivatives phenprocoumon, hymecromone, and psoralen possess better binding efficacy toward at least three targets out of four targets studied when compared to their parent compounds. The results for these compounds indicate lipophilic, high gastrointestinal absorbable, and blood–brain barrier permeability of the compounds studied.^[^ ^227^ ^]^


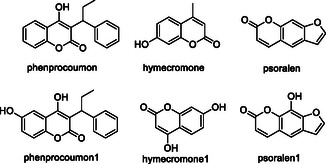


## 11 Conclusions

Drugs used in therapy nowadays are designed to interact with a single, highly selective biological target while minimizing adverse effects. However, it is now well established that the progression of complex diseases, such as cancer and neurological disorders, involves multiple mechanisms. This realization has highlighted the limitations of highly selective single‐target drugs in effectively treating such conditions. To address these challenges, pharmacological agents are increasingly administered in combination rather than targeting a single biological entity. Despite this approach, drug–drug interactions and increased costs have hindered the achievement of optimal therapeutic outcomes. In response, research has focused on developing drugs capable of modulating multiple biological targets within a disease. Studies indicate that targeting multiple pathways can prevent the progression of complex diseases, including Parkinson's disease, AD, and cancer. Moreover, the use of single multitarget drugs has been reported to mitigate the risk of drug–drug interactions, reduce economic burdens, and minimize undesirable biological effects on patients.

Multitarget drugs hold significant potential for treating complex and multifactorial diseases, as well as drug‐resistant conditions. Research groups employing innovative methodologies, such as network analysis, to identify key targets involved in disease pathophysiology and resistance mechanisms anticipate that multitarget drugs will offer improved safety and efficacy compared to single‐target therapies. The core structure of these drugs often consists of heterocyclic scaffolds, which are known for their potent activity against specific targets. However, achieving a balance between desired therapeutic effects and enhanced bioavailability remains a major challenge in multitarget drug design. While increasing the likelihood of success in early‐stage drug development remains a critical goal, and multitargeted drugs hold great promise, significant obstacles must still be overcome on the path to their clinical application.

In conclusion, embracing the principles of polypharmacology is essential to advance the development of effective multitarget drugs for complex diseases. Polypharmacological agents provide a promising strategy to overcome the limitations of highly selective single‐target drugs, including drug resistance and insufficient efficacy. Future research focusing on the integration of polypharmacological principles with novel synthetic methodologies and computational tools will likely accelerate the discovery of next‐generation therapeutics.

## Abbreviations

**Table:** 

ADMET	Absorption, Distribution, Metabolism, Elimination, and Toxicity

## Conflict of Interest

The authors declare no conflict of interest.
